# An extended network for regulation of heme homeostasis in cells

**DOI:** 10.1073/pnas.2508237122

**Published:** 2025-09-30

**Authors:** Andrea E. Gallio, Noa A. Marson, Kate J. Heesom, Philip A. Lewis, Dominic Alibhai, Celyn A. Dugdale, Andrew Herman, Jaswir Basran, Andrew J. Hudson, Emma L. Raven

**Affiliations:** ^a^School of Chemistry, University of Bristol, Bristol BS8 1TS, United Kingdom; ^b^Proteomics Facility, Faculty of Life Sciences, University of Bristol, Bristol BS8 1TD, United Kingdom; ^c^Wolfson Bioimaging Facility, Faculty of Life Sciences, University of Bristol, Bristol BS8 1TD, United Kingdom; ^d^Flow Cytometry Facility, School of Cellular and Molecular Medicine, University of Bristol, Bristol BS8 1TD, United Kingdom; ^e^Leicester Institute for Structural & Chemical Biology, University of Leicester, Leicester LE1 7HB, United Kingdom; ^f^School of Chemistry, University of Leicester, Leicester LE1 7RH, United Kingdom

**Keywords:** tetrapyrroles, heme, heme biosynthesis, biosensing, proteomics

## Abstract

Iron-bound tetrapyrroles (hemes) are essential for the regulation of cellular functions and bioenergetics. The processes of heme biosynthesis, transport, and degradation are responsible for the supply of heme in mitochondria and its insertion into other downstream proteins. What remains unresolved is how these processes interconnect and the wider implications for the cell in the restoration of homeostasis when heme concentrations change. We demonstrate a wide-ranging and coordinated response to changes in intracellular heme in HEK293 cells through a network of complementary mechanisms that extend well beyond the direct regulation of heme biosynthesis and degradation. These responses connect changes in heme homeostasis to mitochondrial function, including core metabolic processes such as the tricarboxylic acid cycle and oxidative phosphorylation, as well as to enzymes involved in the control and storage of iron. Our findings demonstrate far-reaching consequences to perturbations of heme homeostasis and provide insights into the complexity of the cellular hemome.

## Significance

Heme is essential for aerobic life. Heme is synthesized in mitochondria, but the mechanisms that control how heme moves around in cells, and how this connects to the wider aspects of cellular function, are unknown. In this work, we identify a wide-ranging regulatory network that connects heme biosynthesis and degradation with the regulation of iron biology, energy metabolism and oxidative phosphorylation, and mitochondrial health. What emerges is a picture of a dynamic “hemome” linking the control of heme homeostasis to the overall metabolic function of the cell. The results provide the basis for an improved understanding of how disruption of heme homeostasis affects cellular physiology and may impact treatment of diseases linked to tetrapyrrole dysfunction.

Tetrapyrroles are the most abundant family of natural pigments in the biological world. They are famously characterized by their wide range of colors, that has led them to be referred to as “the pigments of life” (1–3). The distribution of tetrapyrroles in nature is ubiquitous. They consist of four differently substituted pyrroles connected either in a cyclic structure or in linear form (the latter known as bilins). Cyclic tetrapyrroles can complex to a number of different metals (e.g., Fe, Co, Mg, Ni, Zn) in the center of the cavity, and these molecules are essential in metabolic, biosynthetic, respiratory, light-harvesting, and regulatory pathways (4–9).

Iron-bound cyclic tetrapyrroles are commonly referred to as hemes. The family of heme-containing proteins is vast, and heme proteins are responsible for a multitude of catalytic and noncatalytic functions that are essential for the survival of almost all organisms. Well-known functions of heme proteins include electron transfer, respiration, and oxygen transport, and oxidative heme enzymes are central to many metabolic pathways in biology (10–16). Recently, however, this decades-long picture of the role of heme in biology has needed revision, in the light of a persuasive body of evidence that demonstrates heme is a primary or secondary regulator of other complex processes in cells. These newly discovered roles for heme include transcriptional regulation of the circadian clock, control of the immune response, neurodegeneration and aging, gas sensing, and the gating of ion channels (4, 7–9, 17–22).

**Fig. 1.**
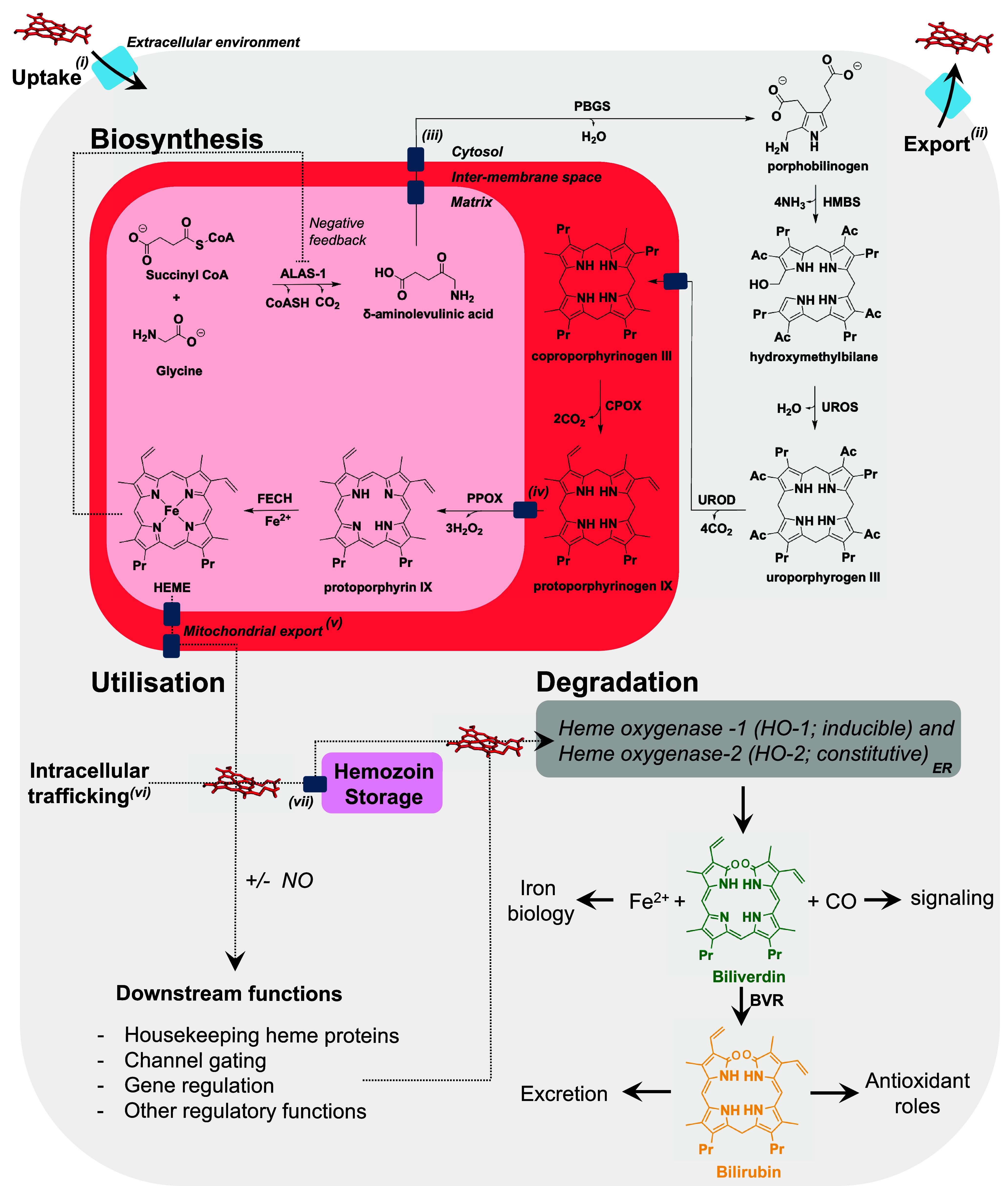
Overview of nonerythroid eukaryotic heme homeostasis. Simplified schematic of a eukaryotic cell, including key intracellular compartments involved in heme homeostasis. The cytosol (light gray), mitochondrion [red(s)], lysosomes (pink), and endoplasmic reticulum (ER, dark gray) are shown. Transmembrane transporters are shown as dark blue rectangles. Annotations refer to proteins involved in tetrapyrroles and heme trafficking where known or proposed: (i) HRG1, FLVCR2, SLC46A1, or endocytosis of circulating heme-bound proteins (e.g. hemopexin, albumin, hemoglobin); (ii) ABCG2, MRP5, FLVCR1a; (iii) ABCB10; (iv) Tmem14c; (v) FLVCR1b; (vi) GAPDH, PGRMC2, TANGO2, buffering proteins; (vii) HRG-1. Note that the list is not exhaustive and only a subset may be applicable to different tissues. Comprehensive reviews are available (27, 28). Heme biosynthesis occurs through an 8-step enzymatic pathway and is partitioned between the mitochondria and the cytosol with the mitochondrial heme biosynthesis proteins forming the heme metabolon δ-aminolevulinate synthase 1 (ALAS-1), porphobilinogen synthase (PBGS), hydroxymethylbilane synthase (HMBS), uroporphyrinogen synthase (UROS), uroporphyrinogen decarboxylase (UROD), coproporphyrinogen oxidase (CPOX), protoporphyrinogen oxidase (PPOX), protoporphyrin ferrochelatase (FECH) (29). Dedicated mitochondrial transporters translocate intermediates. Once formed, heme is exported from the mitochondria for trafficking and intracellular utilization. Nitric oxide (NO, *Bottom Left*) is gaining increasing recognition for its roles in heme mobilization and transfer between heme-binding partners (30). The acidic inner lysosomal environment facilitates heme storage as hemozoin. Heme oxygenase proteins (HO-1/2), primarily tethered to the ER, are responsible for heme degradation. Biliverdin and bilirubin, the linear tetrapyrrole products of heme degradation, aid the maintenance of intracellular redox balance biliverdin reductase (BVR).

For heme to have such wide-ranging control over so many different cellular processes, it follows that the supply of heme and its distribution around the cell must be highly coordinated, to respond to levels of demand and to ensure timely availability of the tetrapyrrole. The supply of heme is dependent on the processes that control heme biosynthesis (an 8-step enzymatic process), heme trafficking, and heme degradation (catalyzed by heme oxygenase enzymes). An overview of these processes, and how they connect to what is currently known about the wider aspects of cellular function, is provided in Fig. 1. What remains unclear is how alterations in the supply of heme affect the overall metabolic landscape of the cell. Understanding these processes is potentially transformative, because opportunities for therapeutic interventions would arise in disorders and fundamental biological processes linked to the disruption of tetrapyrrole homeostasis [e.g., in neurodegeneration, COVID-19, in cardiovascular diseases, and the mechanisms of RNA silencing (23–26)].

To address this challenge, we examine how alterations in intracellular heme levels affect the balance between heme biosynthesis and degradation, and how this radiates out across the cell. The results demonstrate a timely, wide-ranging, and well-coordinated cellular strategy to cope with perturbations in heme concentration that extend far beyond the immediate need for direct regulation of heme synthesis and degradation.

## Results

### Quantification of Cellular Heme Bioavailability.

**Fig. 2.**
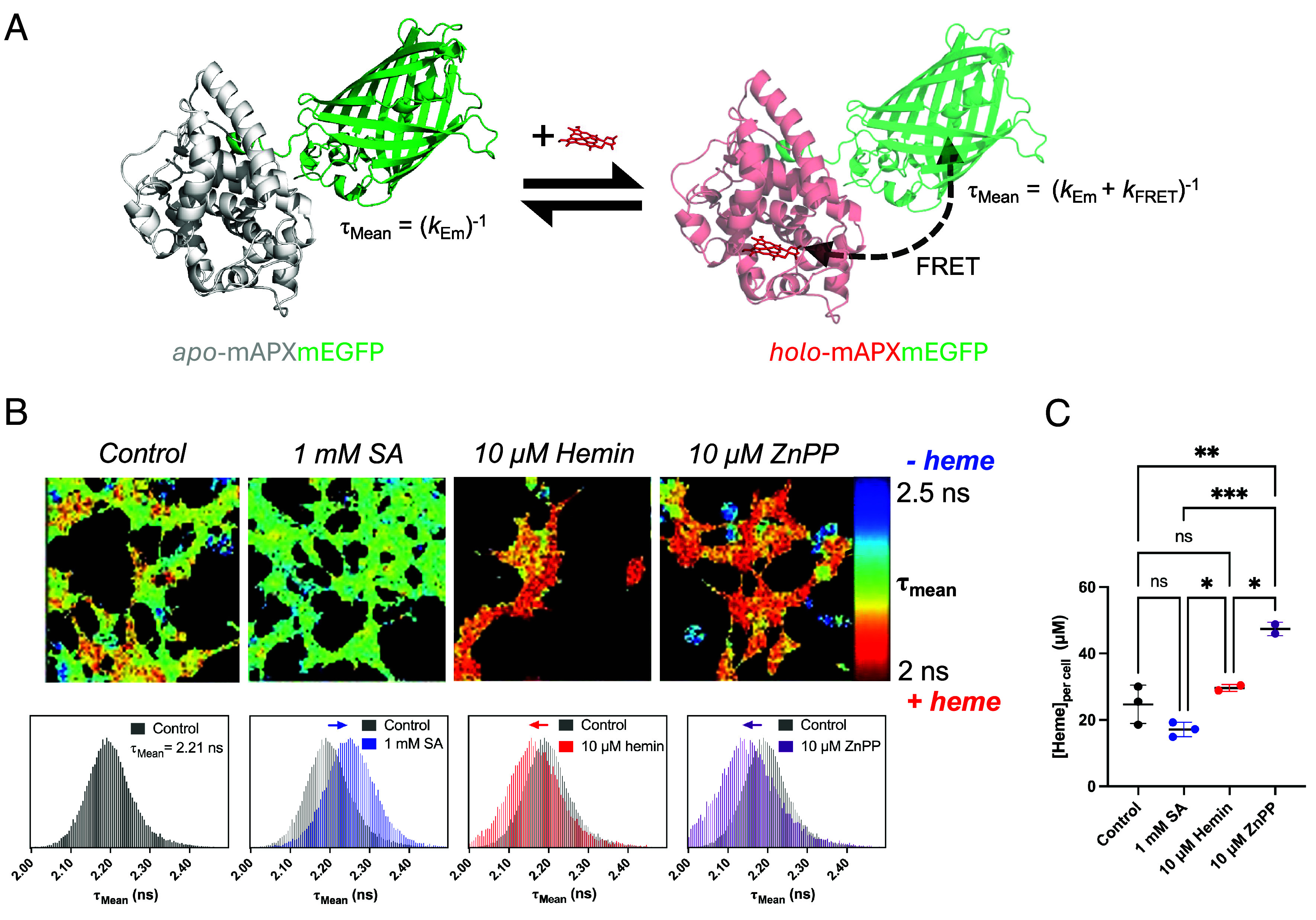
Changes in heme bioavailability. (*A*) Design principle of the heme sensor, mAPXmEGFP. Heme binding to *apo*-mAPXmEGFP to form *holo*-mAPXmEGFP shortens the fluorescence lifetime (τ_mean_) via Föster Resonance Energy Transfer (FRET) (31). Spectral properties and purification of recombinant mAPXmEGFP are in *SI Appendix*, Fig. S1 *C* and *D*. (*B*) Representative color maps of intensity-weighted mean fluorescence lifetime (see main text) obtained from HEK293 cells expressing mAPXmEGFP. Cells were incubated in minimum essential medium which was supplemented with SA (1 mM), hemin (10 μM), or ZnPP (10 μM) and incubated for 24 h prior to imaging (control = no additives; full experimental details available in *Materials and Methods*). The histograms underneath each image show the distribution of τ_mean_ and, for each of the different incubations, compared to the control (black histogram). The centroid of the distribution of τ_mean_ for the control is 2.21 ns, 2.25 ns for 10 mM SA, 2.15 ns for 10 μM hemin, and 2.11 ns for 10 μM ZnPP. Extended datasets with multiple images per condition are shown in *SI Appendix*, Fig. S1*A* and complete fitting reports are presented in *SI Appendix*, Fig. S1*B* and Tables S1–S4. The average concentration of mAPXmEGFP in the transfected HEK293 cells was estimated to be 80 nM, *SI Appendix*, Fig. S3, which falls within the expected range for intracellular heme (32). (*C*) Bar chart showing changes in total heme concentration (average value per cell) following 24 h incubation with 1 mM SA, 10 μM hemin, or 10 μM ZnPP. Multiple comparisons obtained using one-way ANOVA Tukey’s test (*P* = *0.01; **0.002; ***0.0004). The incubation of cells with ZnPP led to the largest accumulation of intracellular tetrapyrrole. This is also evidenced by qualitatively comparing the color of the pellets of cells from the different experiments, with visibly darker pellets produced following incubation with ZnPP, *SI Appendix*, Fig. S1*E*. The control experiment is for cells cultured with maintenance medium only (*Materials and Methods*).

Using a fluorescently tagged heme peroxidase sensor [mAPXmEGFP (31)], we first sought to establish how the overall bioavailability of heme changes under different cellular conditions. Upon heme binding, the mAPXmEGFP sensor exhibits a decrease in the fluorescence lifetime that can be used to quantify heme availability from cellular images, Fig. 2*A* and *SI Appendix*, Figs. S1–S3. In HEK293 cells, we examined different cell culture conditions in which heme concentrations were varied: i) the biosynthesis of heme is inhibited by addition of succinylacetone (SA), which is an inhibitor of PBGS in the second step of the heme biosynthesis pathway; ii) the concentrations of heme are supplemented by addition of hemin; iii) the degradation of heme is inhibited by addition of Zn-protoporphyrin IX (ZnPP), which is a potent inhibitor of heme oxygenase-1 (HO-1) and heme oxygenase-2 (HO-2). For each condition, fluorescence lifetime imaging microscopy (FLIM) revealed the spatial distribution of emission lifetime for the sensor in the cell. The broad distribution of values of the mean fluorescence lifetime, τ_mean_, for each condition reflects differences in local heme availability, Fig. 2*B*. The shift in the modal value of τ_mean_ between the images obtained for different incubations indicates cell-wide changes in bioavailable heme, *SI Appendix*, Fig. S1*B*. This indicates that lower heme levels are observed in the case of cells incubated with SA (up-shift in the distribution of τ_mean_). Conversely, higher heme levels are observed when cells are supplemented with either hemin or ZnPP (down-shift in the distribution of τ_mean_), Fig. 2*B*. This analysis, measured at a single time point for each of the conditions used, is important for comparative purposes but does not take into account the potentially distinct but poorly quantified timescales over which each treatment affects the cellular response. This means that primary (i.e., as a direct result of the addition of SA, heme, or ZnPP) versus secondary (e.g., oxidative stress cascades caused by reactive oxygen species as a direct result of heme addition) effects are not uncoupled.

### Measurement of Total Cellular Heme.

The formation of *holo*-mAPXmEGFP, Fig. 2*A*, depends on a proportion of the total intracellular heme being available for binding to the sensor. We and others consider this to be in the form of “exchangeable” or “bioavailable” heme, which is envisaged to be loosely bound within the cell and available for exchange between binding partners. Exchangeable heme is a subset of the total amount of heme that exists in the cell: While some of the heme is bound irreversibly to heme proteins or stored as hemozoin (33) (and so not available for binding to the sensor), the exchangeable or bioavailable heme is available for regulatory or signaling function (32, 34). To probe whether the measured changes in bioavailable heme shown in Fig. 2*B* were accompanied by similar changes in total heme, we measured the concentration of total intracellular heme ([Heme]_per cell_) using a fluorescence assay (35, 36). Measurements were made in cells incubated with SA, hemin, or ZnPP, Fig. 2*C*; these data reveal concentrations of total heme that are at up to four orders of magnitude higher than those for bioavailable heme reported in several different cell lines (32). A significant increase in total intracellular heme was observed in cells incubated with hemin or ZnPP, compared to cells in which heme biosynthesis was inhibited with SA. Accumulation of total heme was particularly enhanced by incubation with ZnPP, consistent with the inhibition of HO-1 and HO-2 by ZnPP. However, this was not accompanied by the accumulation of bioavailable heme, Fig. 2*B*, suggesting the existence of mechanisms controlling bioavailable heme levels that operate separately from those dedicated to the management of total heme levels.

### Quantification of the Reciprocal Regulation of ALAS-1 and HO-1.

**Fig. 3.**
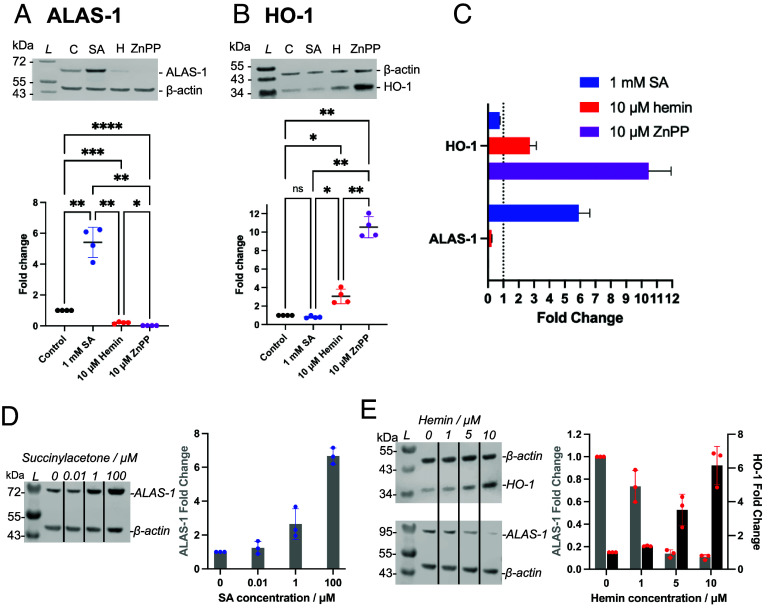
Control of heme biosynthesis and degradation. (*A*) Immunoblot data showing levels of ALAS-1 in whole lysates of HEK293 cells cultured for 24 h with 1 mM SA, 10 μM hemin, or 10 μM ZnPP prior to lysis. (*B*) Measured levels of HO-1 in the same experiment as in (*A*). Representative immunoblots showing a comparison between the band intensities are shown above each chart in (*A* and *B*). Band intensities were measured by densitometry and normalized using β-actin detection as an internal control. Error bars show the SD for n = 4. Normalized fold changes are shown relative to the control. Multiple comparisons obtained using one-way ANOVA Tukey’s test (*P* = *0.025; **0.05; ***0.0004; ****<0.0001). The levels of two other proteins were measured: HO-2, the constitutively expressed heme oxygenase, and GAPDH which is a proposed heme chaperone and buffering molecule (38). No significant changes were observed in the levels of either HO-2 or GAPDH, *SI Appendix*, Fig. S4. (*C*) Bar chart showing the reciprocal relationship of expression levels for ALAS-1 and HO-1 based on the data in (*A* and *B*). Data and representation of the measured fold changes of HO-2 and GAPDH for each treatment are shown in *SI Appendix*, Fig. S4. (*D* and *E*) Representative western blot images for whole cell lysates of HEK293 cells incubated for 24 h with different concentrations of (*D*) SA (0.01 to 100 μM) and (*E*) hemin (1 to 10 μM) prior to lysis. Error bars show the SD for n = 3. The experiments in (*D* and *E*) simulate conditions of increasing heme deprivation (SA) and heme overabundance (hemin) respectively, and further highlight the reciprocal relationship in expression levels of HO-1 and ALAS-1. The changes are compared to an untreated control.

The changes in heme levels observed under different cellular conditions, Fig. 2, need first to be understood within the context of the regulation of heme biosynthesis and degradation. Because a reciprocal feedback regulation of heme biosynthesis and degradation is known to exist (37), we assessed the expression of ALAS-1 and HO-1 as a way of decoupling the overall cellular response. ALAS-1 catalyzes the condensation of glycine with succinyl-CoA (Suc-CoA) to form δ-aminolevulinic acid and is the first and rate-limiting enzyme in the heme biosynthesis pathway, Fig. 1. HO-1 is the inducible form of heme oxygenase which catalyzes the degradation of excess heme, Fig. 1. Whole cell lysates of HEK293 cells were used to quantify the relative changes in levels of ALAS-1 and HO-1 under varying conditions of heme availability (as above), Fig. 3 *A* and *B*. Under conditions where heme biosynthesis is inhibited using SA, ALAS-1 is upregulated, but there is no significant effect on levels of HO-1, Fig. 3 *A* and *B*. Conversely, in the presence of hemin ALAS-1 is down-regulated and HO-1 is up-regulated. The up-regulation of HO-1 is even more pronounced in the presence of ZnPP which also determined a greater loss of ALAS-1 expression, Fig. 3 *A*–*C*. These data show that the ALAS-1 and HO-1 proteins work together to respond to changes in intracellular heme concentration, Fig. 3*C*.

In further experiments, we demonstrated that the expression level of ALAS-1 and HO-1, as shown above, are responsive to a range of concentrations of SA and hemin, Fig. 3 *D* and *E*. With increasing concentrations of SA (0.01 to 100 μM), ALAS-1 levels exhibited a monotonic increase, Fig. 3*D*. With increasing concentrations of hemin (1 to 10 μM), a monotonic decrease in ALAS-1 accompanied by a monotonic increase of HO-1 was observed, Fig. 3*E*. Together, these data demonstrate a dynamic interplay between the key heme homeostasis proteins that are directly responsible for heme synthesis and heme degradation, and that this interplay is responsive to changing concentrations of bioavailable heme.

### Quantitative Proteomics.

**Fig. 4.**
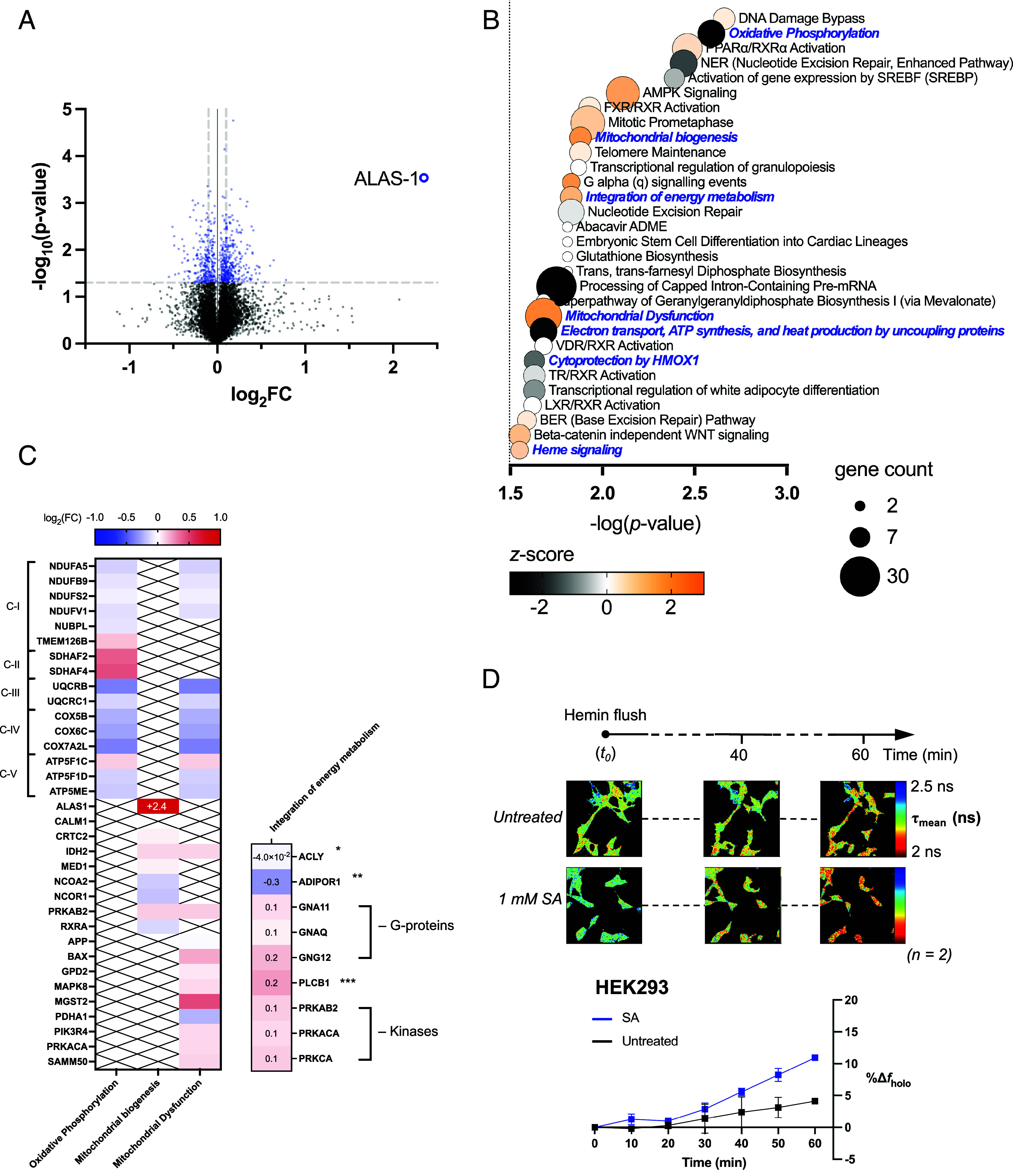
Inhibition of heme biosynthesis induces mitochondrial dysfunction, inactivates oxidative phosphorylation, and increases extracellular heme uptake. (*A*) Volcano plot showing the global changes in protein levels in HEK293 cells incubated for 24 h with 1 mM SA relative to whole cell lysates of control cells. The –log_10_(*P*-value) of each protein was plotted against the log_2_(FC) (FC: fold change). Differentially expressed proteins with *P*-value < 0.05 are shown in blue. The up-regulation of ALAS-1 is highlighted. (*B*) Bubble plot of the biological pathways affected by the inhibition of heme biosynthesis. IPA was applied to the TMT-proteomics data shown in (*A*). The color scale represents the *z*-score, a statistical parameter assigned for the activation or inactivation of a given pathway. Pathways with *z*-score > 2 or *z*-score < −2 are predicted by IPA to be either strongly activated or inactivated, respectively. For clarity, only pathways for which −log(*P*-value) > 1.5 are shown. Pathways directly related to mitochondrial processes or heme biology are shown in blue. The complete list of biological pathways identified by IPA through incubation with 1 mM SA is shown in *SI Appendix*, Fig. S6. (*C*) The heatmap on the *Left* shows a list of mitochondrial genes as grouped by IPA according to a specific mitochondrial pathway or process: oxidative phosphorylation, mitochondrial biogenesis, and mitochondrial dysfunction. Genes affecting oxidative phosphorylation are grouped by brackets according to the complex they belong to within the pathway. The color scale reports the measured log_2_FC in (*A*). The activation of signaling pathways for the integration of the energy metabolism were highlighted by IPA. The heat-map on the *Right* shows the measured log_2_(FC) of proteins involved in this pathway such as G-proteins and kinases that are regulators of glucose and lipid metabolism, ATP-citrate synthase and the ADIPOQ-receptor (*ATP-citrate synthase provides substrates for de novo cholesterol and fatty acids synthesis; **ADIPOQ receptor; ***DAG/IP3 hydrolase mediates signaling of G protein–coupled receptors). (*D*) Time-series FLIM of clusters of HEK293 cells expressing mAPXmEGFP. Immediately prior to imaging, hemin was added directly to cells cultured in microscopy dishes (final concentration 10 μM). One frame was acquired every 10 min for a duration of 1 h. Decreasing values of τ_mean_ for mAPXmEGFP over time indicate real-time heme uptake. Separate experiments were carried out by adding hemin to cells incubated 24 h in MEM only (control, *Top* row) and cells incubated for 24 h in MEM supplemented with 1 mM SA (*Bottom* row). From the FLIM data, the fraction of *holo*-mAPXmEGFP corresponding to each time point can be quantified and expressed as percentage change relatively to *t*_0_ (%Δ*f*_holo_, see *SI Appendix*). Error bars show the SD for n = 2. Comparison between untreated (black trace) and SA-incubated cells (blue trace) shows enhanced heme uptake when heme biosynthesis is inhibited, highlighting the adaptive mechanisms in cells to ensure adequate heme supply.

**Fig. 5.**
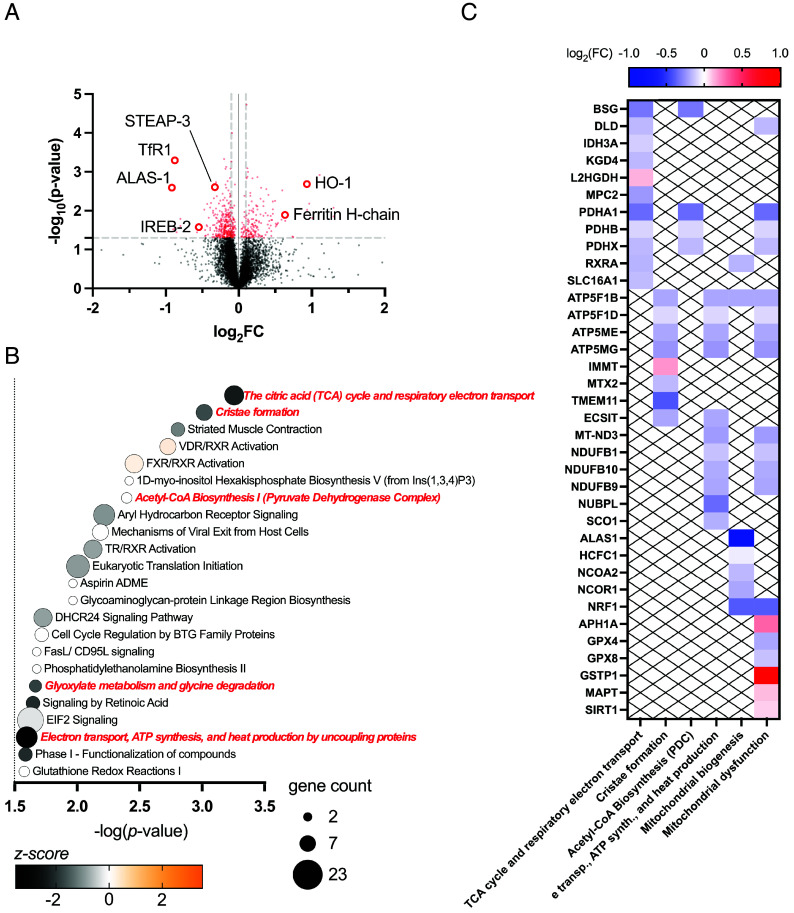
Incubation with hemin inactivates the TCA cycle and affects iron biology. (*A*) Volcano plot showing the global changes in the protein levels in HEK293 cells incubated for 24 h with 10 μM hemin relative to whole cell lysates of a control. The –log_10_(*P*-value) of each protein was plotted against the log_2_(FC) (FC: fold change). Differentially expressed proteins with *P*-value < 0.05 are shown in red. The upregulation of HO-1 and the down-regulation of ALAS-1 are highlighted. Also highlighted are the proteins involved in iron uptake, transport, and storage (STEAP-3, TfR1, IREB-2, and ferritin H-chain). Under conditions of high heme, the up-regulation of HO-1 leads ultimately to release of iron from heme, an increased requirement for iron storage (by ferritin), and a decreased requirement for iron uptake (STEAP-3 and TfR). (*B*) Bubble plot of biological pathways significantly activated or inactivated as predicted by IPA based on the data shown in (*A*). Assigned *z*-scores are indicated by the color scale underneath the bubble plot. For clarity, only pathways for which −log(*P*-value) > 1.5 are shown. Pathways directly related to mitochondrial processes or heme biology are shown in red. The complete list of biological pathways identified by IPA through incubation with 10 μM hemin is shown in *SI Appendix*, Fig. S7. (*C*) The heatmap shows a list of mitochondrial genes as grouped by IPA according to a specific mitochondrial pathway or process: TCA cycle and respiratory electron transport, cristae formation, acetyl-CoA biosynthesis mediated by the PDC, electron transport, ATP synthesis, and heat production by uncoupling of proteins, mitochondrial biogenesis, and mitochondrial dysfunction. To the best of our knowledge, none of the indicated protein subunits uses heme as cofactor. The color scale reports the measured log_2_FC in (*A*).

We hypothesized that the processes for heme biosynthesis and degradation are unlikely to operate in isolation. We therefore sought to identify more wide-ranging consequences of changes in intracellular heme levels than those shown in Figs. 2 and 3. Whole cell lysates of HEK293 cells that had been incubated with 1 mM SA, 10 μΜ hemin, or 10 μM ZnPP in the same way as above, were analyzed using Tandem Mass Tag (TMT) proteomics. The numbers of proteins that were significantly up- or down-regulated under each condition are shown in *SI Appendix*, Fig. S5. The most immediate observation from these data is that the pattern of up- and down-regulation of ALAS-1 and HO-1 observed by immunoblotting, Fig. 3, is reproduced in the quantitative proteomics analyses, Figs. 4*A* and 5*A* and *SI Appendix*, Fig. S8*A*, thus validating the approach. Using the Ingenuity Pathway Analysis (IPA, see *SI Appendix*), it was possible to identify in the proteomics data wider relationships between bioavailable heme levels and cellular metabolic function molecules. IPA compares the list of significantly changed proteins to the full list of detected proteins and identifies whether clusters of proteins associated with particular functions or pathways are significantly enriched. IPA further uses the relative changes in protein abundance in these clusters to predict activation or inhibition. IPA outputs of significantly altered biological pathways (*P* < 0.05) are summarized for incubations with SA (Fig. 4*B*), hemin (Fig. 5*B*), and ZnPP (*SI Appendix*, Fig. S8*B*), respectively. Notably, both SA and hemin affected several mitochondrial processes related to energy production, as presented below.

In the case of cells incubated with SA to inhibit heme synthesis, we identified a down-regulation of NADH dehydrogenase subunits and several other proteins involved in electron transport processes, including cytochrome *c* oxidase subunits, cytochrome *b*_5_, and the cytochrome *bc*1 complex, Fig. 4*C*. This is consistent with an overall inactivation of oxidative phosphorylation, Fig. 4*B*. This disruption of core metabolic processes under conditions where heme synthesis was inhibited led to mitochondrial dysfunction and was accompanied by the activation of mitochondrial biogenesis as well as activation of pathways involved in the integration of energy metabolism, Fig. 4 *B* and *C*. We interpret this to mean that depletion of bioavailable heme in the presence of SA negatively impacts fundamental heme-dependent mitochondrial processes (e.g., oxidative phosphorylation) and activates auxiliary modes of energy production (mitochondrial biogenesis and integration of energy metabolism).

Given the inactivation of oxidative phosphorylation as a consequence of inhibition of heme biosynthesis, we sought to measure whether cells could subsequently compensate for this with an increased capacity for extracellular heme uptake. When control cells expressing mAPXmEGFP are treated with hemin immediately before imaging, τ_mean_ decreases over a period of 60 min, which is consistent with uptake of hemin into cells, Fig. 4*D*. When cells are preincubated with SA, there is a more dramatic decrease in the modal value of τ_mean_ after addition of hemin, which is consistent with an overall higher uptake of extracellular heme. In a set of parallel experiments, HeLa cells were also preincubated with SA in the same way, and a similar pattern was obtained, *SI Appendix*, Fig. S9. However, HeLa cells showed consistently higher heme uptake after 60 min (compare Fig. 4*D* and *SI Appendix*, Fig. S9), indicating that heme uptake capacity varies in different cell types, presumably reflecting distinct metabolic profiles and heme demands, *SI Appendix*, Fig. S9.

Turning to the effects of increased heme, several mitochondrial processes were also affected by incubation with hemin, including the tricarboxylic acid (TCA) cycle and mitochondrial biogenesis as shown in Fig. 5 *B* and *C* and *SI Appendix*, Fig. S7. The TCA cycle was found to be significantly inactivated through a down-regulation of isocitrate dehydrogenase (IDH3A) and α-ketoglutarate dehydrogenase component 4 (KGD4), both catalyzing the conversion of TCA cycle intermediates preceding the formation of Suc-CoA, and the down-regulation of PDHA1, PDHB, and PDHX subunits of the pyruvate dehydrogenase complex (PDC), which converts aerobically generated pyruvate into acetyl-CoA entering the TCA cycle, Fig. 5*C*. As in the case of cells incubated with SA (above), incubation with hemin also affected mitochondrial dysfunction, Fig. 4*B* and *SI Appendix*, Figs. S6 and S7. However, mitochondrial biogenesis was in this case inactivated and accompanied by a disruption in the formation of mitochondrial cristae, marked by the down-regulation of several ATP synthase subunits and the up-regulation of Mic60 (IMMT), a core component of the mitochondrial contact site and cristae organizing system (MICOS), Fig. 5 *B* and *C*. MICOS plays a crucial role in facilitating ferrochelatase activity, essential for heme biosynthesis (39). The data also identify a direct effect on iron uptake and storage as a consequence of incubation with hemin. In particular, the heavy chain of the iron storage protein ferritin was up-regulated, while two proteins involved in the iron acquisition pathway, STEAP-3 and the transferrin receptor (TfR), were down-regulated, Fig. 5*A*. Finally, given the known interplay between hemin toxicity and glutathione (GSH) depletion, changes in thiol redox status may contribute to the observed proteomic shifts (40, 41). Our pathway analysis identified proteins involved in glutathione biosynthesis (Fig. 4*B*) and glutathione redox reactions (Fig. 5*B*). While this supports the concept of possible cross talk between heme and GSH, it was not possible from the current data to determine whether these pathways were activated or inactivated. A direct comparison of pathways that were significantly affected by both incubations with hemin and SA is provided in *SI Appendix*, Fig. S10.

Of the three conditions examined, the ZnPP incubation resulted in the highest number of significantly affected proteins (*SI Appendix*, Fig. S5). ZnPP exhibits a similar effect to that induced by hemin on heme biosynthesis and degradation. However, unique to ZnPP is an up-regulation of PPOX and FECH, two mitochondrial enzymes catalyzing the last two steps of heme biosynthesis, *SI Appendix*, Fig. S8*A*. Compared to hemin, the measured up-regulation of HO-1 is more pronounced (Figs. 3 *A*–*C* and 5*A* and *SI Appendix*, Fig. S8*A*). The inhibitory effect of ZnPP on heme oxygenase activity prevents HO-1 from alleviating intracellular stress (42, 43) and leads to heme accumulation, Fig. 2*C*, both consistent with the greater HO-1 induction in comparison to hemin. It therefore follows that the impact of ZnPP extends across additional oxidative stress responses. These stress responses are mainly mediated by NRF2-target genes which includes BACH1 [which is heme-dependent (44)] and glutathione S-transferase P1 (GSTP1). Multiple other pathways, consistent with responses designed to support cell survival under stress, were also identified. The list includes the down-regulation of NRF1, gene silencing by RNA, posttranslational modifications (e.g., SUMOylation, ubiquitination), and activation of metabolic pathways such as NAD signaling and mitochondrial fatty acid β-oxidation, *SI Appendix*, Fig. S8*B*.

### Assessment of Mitochondrial Function.

The effects of changes in heme concentration on mitochondrial processes, as evidenced above were further assessed using the 3-(4,5-dimethylthiazol-2-yl)-2,5-diphenyl-2H-tetrazolium bromide (MTT) assay, *SI Appendix*, Fig. S12*A*. The MTT assay exploits the chromogenic nature of the reduction of a mono-tetrazolium salt to formazan by metabolically active cells, and is used to assess changes in overall cellular metabolic activity, especially in relation to mitochondrial function (45). Decreased formazan production was measured following incubations with SA, hemin, or ZnPP, indicating compromised mitochondrial activity and with the greatest effect observed with hemin and ZnPP, *SI Appendix*, Fig. S12*A*, consistent with our proteomics findings.

We further deployed flow cytometry to establish whether the observed changes in mitochondrial metabolic activity were accompanied by altered mitochondrial mass (*SI Appendix*). Mitochondrial staining was used to measure Median Fluorescence Intensities (MFIs) and evaluate relative changes in mitochondrial mass for each incubation condition, *SI Appendix*, Figs. S11 and S12 *B* and *C*. No significant change was observed following inhibition of heme biosynthesis with SA. On the other hand, hemin and ZnPP induced a 20 to 30% drop in measured MFI compared to the control. These data show that the accumulation of intracellular tetrapyrroles can decrease mitochondrial mass.

## Discussion

Over the past decade, progress has been made in tracking both locations and concentrations of heme in cells using fluorescent reporters (31, 44, 46–52). This has shed light on intracellular heme concentrations in isolated examples at a single point in time and has revealed that there are significant variations across different cell lines (with reports varying from low nM to mM, reviewed in refs. 32 and 53). These differences point to the availability of heme being highly dynamic and responsive to the immediate needs of the cell. What is clear is that fluorescent heme reporters, in isolation, cannot yet capture the complexity of this ever-changing local cellular environment. In this paper, we have therefore taken a different approach by examining how perturbations in the abundance of heme are controlled, and how these responses are connected to the wider maintenance of cellular balance.

The first observation is that fluorescence lifetime imaging data provide evidence for fluctuations in local heme bioavailability under conditions where heme concentrations were expected to decrease (when heme biosynthesis was inhibited) or increase (when cells were supplied with heme or when heme degradation was inhibited), Fig. 2. However, only cells incubated with ZnPP showed a significant accumulation of total heme (defined as the amount of heme that is both reversibly and irreversibly bound to heme-binding proteins in the cell), Fig. 2*C*. This means that, in our experiments, changes in bioavailable heme under different conditions of heme exposure do not necessarily affect the total quota of heme in the cell. The substantive conclusion arising from these data is that there is a dynamic response of the cell to changes in the abundance of heme and that different layers of control exist for the maintenance of appropriate levels of overall heme. Part of this overall control is provided by the complementary response of ALAS-1, which supplies heme, and HO-1, which removes it—meaning that there is a tightly coupled coordination between the processes of heme biosynthesis and heme degradation, with ALAS-1 and HO-1 working cooperatively as natural sensors for, and regulators of, intracellular heme levels.

Going beyond the immediacy of the regulation of heme biosynthesis and degradation, we identify more wide-ranging responses to changes in heme homeostasis. The data identify direct connections between fluctuations in heme abundance and a range of proteins involved in mitochondrial function, energy metabolism, and iron regulation, demonstrating that heme homeostasis is integrated with other core pathways. Specifically, depletion of bioavailable heme led to mitochondrial dysfunction and impaired oxidative phosphorylation by affecting several subunits of the electron transport chain, Fig. 4*C*. This latter effect was compensated for by activation of auxiliary modes of energy production, as seen in Fig. 4*C*, and an increased capacity for extracellular heme uptake, Fig. 4*D*, which we interpret as a response to decreased levels of heme available for processes such as oxidative phosphorylation.

**Fig. 6.**
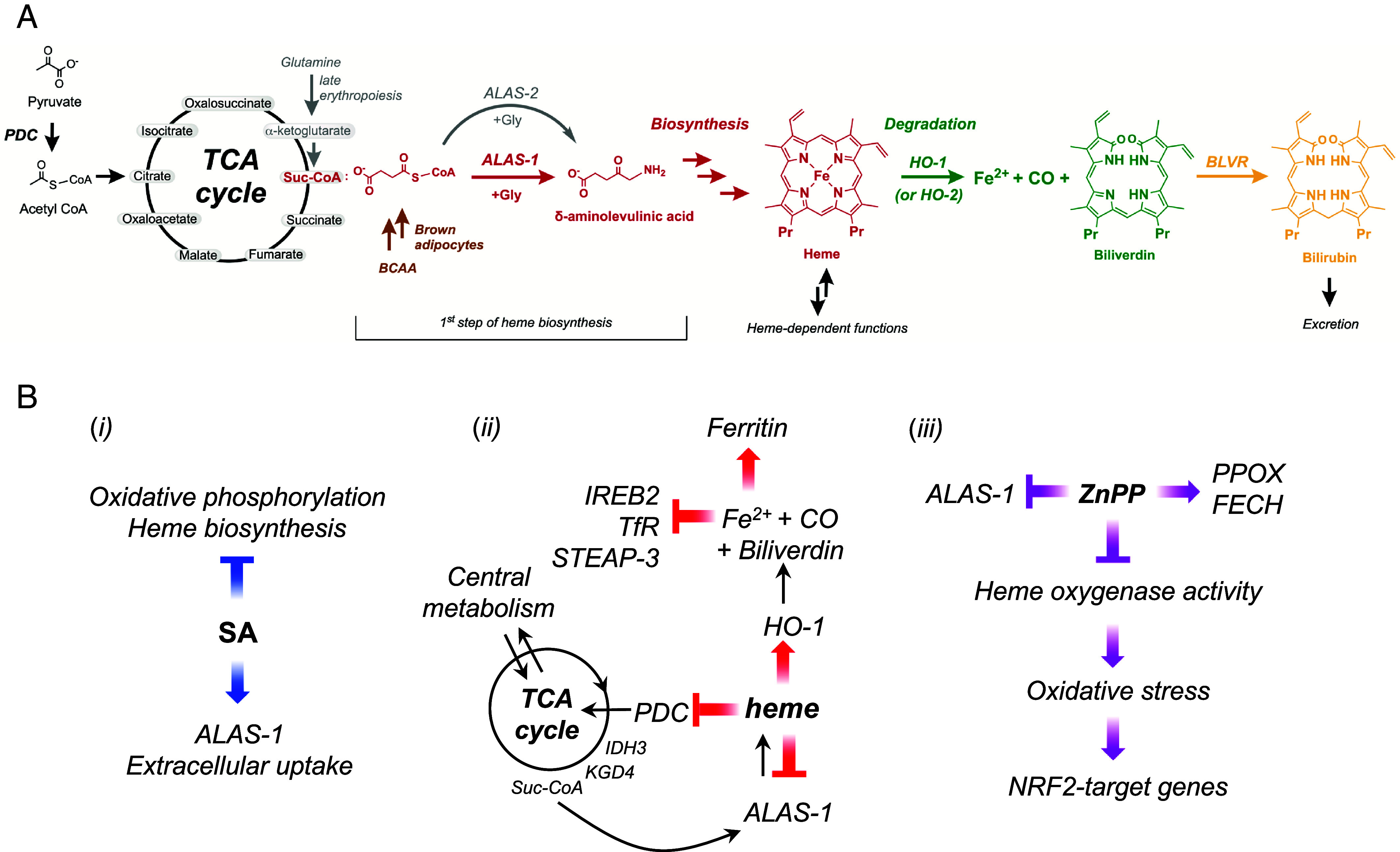
A connected network for control of heme homeostasis. (*A*) Overview of the intracellular metabolism of heme. Intracellular heme is synthesized (in red; Pr: propionate groups) and made available for heme-dependent functions prior to its degradation by heme oxygenase (HO-1 and HO-2; in green). The balance between these two pathways maintains heme homeostasis. The first step of heme biosynthesis—condensation of Suc-CoA and glycine by ALAS1—is dependent on the TCA cycle which produces Suc-CoA. The conversion of pyruvate into acetyl-CoA (shown on the *Left*) by the PDC is a main TCA cycle point of entry during anabolic metabolism. Alternate routes for the provision of Suc-CoA for heme synthesis are shown for red blood cells undergoing the late stages of erythropoiesis (gray) (60), and brown adipocytes (brown; BCAA: branched Chain Amino Acids) (61). Heme degradation leads to the formation of the biliverdin (green) and bilirubin (yellow; BLVR: biliverdin reductase). These linear tetrapyrroles are ultimately excreted but function as key antioxidants. (*B*) Schematic representation of observed responses to changes in heme concentration in HEK293 cells following incubation with SA (blue), hemin (red), or ZnPP (purple). Thick colored arrows show protein up-/down regulation or pathway activation/inactivation, and thin arrows show enzymatic conversions. (*i*) Under conditions of decreased heme, through inhibition of heme biosynthesis with SA, oxidative phosphorylation is inhibited, ALAS-1 is up-regulated, and the capacity for extracellular heme uptake is increased. (*ii*) Under conditions of increased heme, heme biosynthesis and degradation are, respectively, down-regulated (ALAS-1) and upregulated (HO-1). Inactivation of the TCA cycle limits the supply of substrates (Suc-CoA) for heme biosynthesis with the down-regulation of the IDH3 and KGD4 subunits of PDC. The upregulation of HO-1 releases iron [shown in (*A*)] from excess heme, which in turn down-regulates the IREB-2, TfR, and STEAP-3 iron acquisition proteins and up-regulates the iron storage protein ferritin, consistent with a response to iron overload under these conditions. (*iii*) Incubation with ZnPP up-regulates HO-1 but inhibits heme oxygenase activity leading to heme accumulation. The measured effects on heme biosynthesis under these conditions extend beyond the immediate down-regulation of ALAS-1 and include up-regulation of the PPOX and FECH heme synthesis enzymes. Several concomitant pathways consistent with increased levels of oxidative stress were observed, particularly mediated by the BACH1-Nrf2 regulation axis, and also leading to decreased mitochondrial mass (see text). Metabolic maps and a summary table of the main effects determined by each condition in (*i*–*iii*) are available in *SI Appendix*, Fig. S14 and Table S5, respectively.

Perhaps most strikingly, a direct connection between increased heme levels and inactivation of the TCA cycle was identified, Fig. 5 *B* and *C*. Inactivation of the TCA cycle limits ATP production, is associated with metabolic reprogramming, and leads to imbalances in the pools of crucial metabolites for biosynthetic functions [e.g., fatty acid, amino acid, and nucleotide synthesis (54)]. In the context of heme synthesis, the TCA cycle supplies Suc-CoA (a substrate for ALAS-1), and thus, Suc-CoA would become more limited. We interpret this as evidence that heme homeostasis can be controlled by limiting the supply of Suc-CoA for heme biosynthesis. This connection to the TCA cycle emphasizes the centrality of heme for mitochondrial activity and demonstrates modulation of heme biosynthesis in nonerythroid cells by finely tuning the provision of Suc-CoA as a substrate for ALAS-1. We note that a role for Suc-CoA synthetase in regulating heme production was suggested in 1965 (55); later, interactions between Suc-CoA synthetase and ALAS-2 (the erythroid-specific ALAS isoform), but not ALAS-1, were discovered (56). However, recent studies—including this one—highlight a tissue-specific regulation of this interplay, Fig. 6*A* (57). More generally, increased heme levels—both through incubations with hemin and ZnPP—led to a measurable decrease in mitochondrial mass, *SI Appendix*, Fig. S12 *B* and *C*. This is consistent with the pathway analysis, which identifies inactivation of mitochondrial biogenesis and cristae formation in the presence of excess heme, Fig. 5*B* and *SI Appendix*, Fig. S7, leading to fewer functional mitochondria being produced. Overall, a decrease in mitochondrial mass would limit the generation of reactive oxygen species and oxidative stress in the presence of excess heme and high intracellular iron, as evidenced by the emergence of mitigation strategies for iron overload when heme levels increase, Fig. 5. Shifts in intracellular heme levels and oxidative stress are closely linked, with reactive oxygen species known to activate transcription factors such as Nrf2 (NFE2L2) and HIF-1α (58). Incubation with ZnPP led to the activation of these pathways (*SI Appendix*, Fig. S8), consistent with increased oxidative stress due to heme accumulation resulting from the inhibition of HO-1/2. In contrast, incubation with hemin did not significantly alter the Nrf2 and HIF-1α pathways, indicating that the cytoprotective activity of HO-1 can (when functional) mitigate both heme overload and oxidative stress. The transcriptional control of the HO-1 gene is involved in the adaptive cellular response of a wide range of stress conditions that are not limited to increased heme levels (42). The upregulation of HO-1 induced by ZnPP compared to hemin is likely amplified by competition for heme binding sites, heme accumulation caused by inhibition of heme degradation, and increased oxidative stress, likely worsened by limited bilirubin production in the presence of ZnPP (59). Unique to ZnPP is its effect on heme biosynthesis, which extends beyond the down-regulation of ALAS-1 and includes a measurable up-regulation of PPOX and FECH, *SI Appendix*, Fig. S8*A*. It is significant that ALAS-1, PPOX, and FECH are the three mitochondrial enzymes that catalyze their respective steps of the heme synthesis pathway in the mitochondria (with the rest of the heme synthesis pathway occurring in the cytosol). This is consistent with these three mitochondrial heme synthesis enzymes being physically associated with the mitochondrial contact site and cristae organizing system (MICOS) (39), which was shown to be affected by incubation with hemin, Fig. 5*C*. Since hemin affected ALAS-1 and Mic60 (a key MICOS subunit) but not PPOX, and FECH was uniquely influenced by ZnPP and not by hemin, the translational regulatory interactions among the individual components of the mitochondrial heme metabolon appear to be complex. The significance is that these data link fluctuations in the intracellular levels of bioavailable heme with mechanisms that affect mitochondrial function and architecture.

From the overall perspective of cellular homeostasis, the results presented in this study highlight a wide-ranging and well-connected regulatory network that extends far beyond immediate interventions in the amounts of heme that are synthesized or degraded, Fig. 6. Instead, heme homeostasis involves multiple layers of control by integrating its regulation within that of primary metabolic pathways such as the TCA cycle and oxidative phosphorylation, general mitochondrial function, and iron storage and mobilization. Further exploration of this interplay between central metabolism and heme biosynthesis, trafficking, and degradation will, in the future, provide opportunities for interventions in clinical contexts where the dynamics of heme homeostasis is directly disrupted [e.g., porphyrias (62, 63), Chagas disease (64–66), malaria (67, 68)] and/or poorly understood [e.g., cardiovascular physiology (25), and cancer (69–71)].

## Materials and Methods

HEK293 and HeLa cells were purchased from the European Collection of Authenticated Cell Cultures and transfected to stably express mAPXmEGFP. FLIM was performed on a Leica SP8 AOBS confocal laser-scanning microscope attached to a Leica DMi8 inverted epifluorescence microscope. Photon detection was carried out with PicoQuant electronics for Time Correlated Single Photon Counting. For proteomics, cell lysates were digested with trypsin, labeled with Tandem Mass Tag reagents, and processed for nano-LC MSMS analysis. Detailed methods are available in *SI Appendix* for cell culture, FLIM, immunoblotting, total heme assay, proteomics, MTT assay, and flow cytometry.

## Supplementary Material

Appendix 01 (PDF)

Dataset S01 (XLSX)

Dataset S02 (XLSX)

Dataset S03 (XLSX)

Dataset S04 (XLSX)

## Data Availability

All study data are included in the article and/or supporting information.
