# Supplementary material for: An extended network for regulation of heme homeostasis in cells
Source: Proc Natl Acad Sci U S A. 2025 Sep 30;122(40):e2508237122. doi: 10.1073/pnas.2508237122 (PMC12519212; doi:10.1073/pnas.2508237122)
Supplement: Supplementary file 1 — Appendix 01 (PDF) [file pnas.2508237122.sapp.pdf]

PNAS

Supplementary Information for
An extended network for regulation of heme homeostasis in cells.

Andrea E. Gallio, Noa A. Marson, Kate J. Heesom, Philip A. Lewis, Dominic Alibhai, Celyn A. Dugdale, Andrew Herman, Jaswir Basran, Andrew J. Hudson, Emma L. Raven*.

*Corresponding author. Emma L. Raven; School of Chemistry, University of Bristol, BS8 1TS Bristol, UK; Tel: (0117) 455 7999.
Email: emma.raven@bristol.ac.uk.

This PDF file includes:

Materials and Methods
Figures S1 to S14
Tables S1 to S5
SI References

Other supplementary materials for this manuscript include the following:

Data S1 to S4

Materials and Methods

Cell culture. HEK293 and HeLa cells were purchased from the European Collection of Authenticated Cell Cultures and cultured at 37°C and 5% CO_2 in the following maintenance medium: MEM α (Minimum Essential Medium α; Gibco 12561056) containing 10% fetal bovine serum (FBS; Gibco, 10500064) and 1% (v/v) penicillin-streptomycin (Gibco; 15140-122). Medium used in this work was prepared using a single batch of FBS. Total heme content in the complete formulation of maintenance medium was determined equal to 490 nM by total heme assay (see "Analysis of total heme content", below). Cells expressing the fluorescently-tagged mAPXmEGFP peroxidase sensor were supplemented with 200 µg/mL geneticin. Medium was refreshed every 2-3 days and cells were sub-cultured when confluency reached approximately 80% by trypsinization using TrypLE (Gibco, 12604021).

All incubations in the presence of SA, hemin, or ZnPP were carried out for 24 hours prior to cell lysis unless otherwise stated. The culture media containing the respective additives were prepared fresh prior to each incubation by dilution from concentrated aqueous stocks in maintenance medium as above. For medium supplemented with SA, SA was diluted from a stock solution (200 mM) in water. For the preparation of hemin stocks, a micro spatula tip of hemin chloride (Sigma Aldrich) was suspended in 1.5 mL centrifuge tube by adding 50 µL of 0.1 M NaOH (VWR). The suspension was diluted with 1 mL of deionised water and centrifuged (17000 g; 30 s). The supernatant was removed (500 µL) and diluted 1:1 with deionised water in a clean tube. Centrifugation and dilutions were repeated until a clear solution was obtained. The solution of hemin was filtered using a 2 mL Corning Costar Spin-X centrifuge tube with 0.22 µm pores cellulose acetate membrane filter (cls8160). The concentration of the sample was measured from the absorbance at 385 nm, using ε_{385} = 58400 $M^{-1}cm^{-1}$. For ZnPP (Sigma) solutions, 5-10 µL aliquots of a 25 mg/mL DMSO stock were typically diluted 1:1 with 0.1 M NaOH (VWR). The suspension was diluted with 750 µL of deionised water and centrifuged in a tabletop microcentrifuge (17000 g; 1 min). The supernatant was removed (500 µL) and diluted 1:1 with deionised water in a clean tube. Centrifugation was repeated and the supernatant filtered as for the hemin solution detailed above. The absorbance of the sample at 412 nm was measured by UV-Vis spectroscopy using ε_{412} = 87400 $M^{-1}cm^{-1}$.

Generation of stable HEK293 cell line for the expression of mAPXmEGFP. HEK293 cells were seeded in 2 mL culture medium in a polystyrene-coated 6-well plates (Greiner). When confluency of 70-80% was reached, 200-500 ng of pLEICS-138 (PROTEX, University of Leicester) harbouring the sequence for mAPXmEGFP was used for each transfection using Lipofectamine 3000 (Invitrogen) as per the manufacturer's instruction and using serum-free MEM α. After 24-48 h the medium was changed to a formulation containing 10% FBS and cell cultured until confluent. Cells were sub-cultured onto a 10 cm Petri dish for antibiotic screening by refreshing the medium containing a suitable antibiotic every second day over a period of approximatively four weeks. Transfected cells were screened with 500 µg/mL geneticin (G418, Thermo Fischer Scientific). Antibiotic selection was continued until single clones appeared. Clones were isolated using the agarose cloning method and individually sub-cultured for functional tests. Cell stocks of successful clones were prepared by harvesting confluent cultures and resuspending them in maintenance medium as defined above containing 5% DMSO. At least 1 million live cells were aliquoted in 1 mL cryo-tubes and incubated overnight in an *iso*-propanol bath at -80°C. Cryo-tubes were then placed under liquid nitrogen for long-term storage.

Fluorescence Lifetime Imaging Microscopy (FLIM). For the lifetime measurement of mAPXmEGFP, the transfected HEK293 cells were cultured in glass-bottom Greiner Bio-One CELLview advanced TC 35/10 mm dishes (627975) with phenol red free maintenance medium (as above) and supplemented with fresh medium, 1 mM SA, 10 µM hemin, or 10 µM ZnPP at 24 h before imaging. FLIM was performed on a Leica SP8 AOBS confocal laser-scanning microscope attached to a Leica DMi8 inverted epifluorescence microscope (University of Bristol, Wolfson Bioimaging Facility) enclosed in an environmental chamber (Life Imaging Services) for temperature control (set at 37°C) and CO_2 enrichment. For the excitation of mAPXmEGFP, a pulsed white light laser (WLL, 470-670 nm, 2.5 mW/nm, 40 MHz repetition rate) was used. Cell clusters were focused with a 63X/1.20 water immersion lens (63x HC PL APO CS2; serial number: 506361) equipped with a motorised correction collar. A

notch filter centred at 488 nm suppressed laser reflections, and fluorescence emission was collected between 495 and 551 nm (image format: 256×256). Photon detection was carried out with a Leica HyD detector operating in single photon counting and PicoQuant electronics for Time Correlated Single Photon Counting (TCSPC) fluorescence lifetime data acquisition and SymPhoTime software (PicoQuant) (Scan speed: 10 Hz; accumulated frames per image: 3).

Typically, 10 images per condition were acquired (Control, 1 mM SA, 10 µM hemin, or 10 µM ZnPP), with each image focused on a separate cluster of cells. At least two independent biological replicates were measured per condition (Tables S1-S4). A representative set of images for one biological replicate per condition is shown in Figure S1A. Figure S1B shows the measured change in the intensity-weighed mean lifetime, τ_{mean},for all biological replicates.

Fitting of the FLIM data. Time-correlated single-photon counting data in images recorded from HEK293 expressing mAPXmEGFP were fitted globally, using the FLIMfit software (1), to a biexponential decay function, E(t), with set time constants τ_{slow} = 2.7 ns and τ_{fast} = 1.3 ns, Equation S1.

$$E(t) \propto \alpha_{slow}\exp(-t/\tau_{slow}) + \alpha_{fast}\exp(-t/\tau_{fast}) \qquad \text{...Eq. S1}$$

Individual values for the amplitudes of the decay components (α_{fast} and α_{slow}) were used for the calculation of τ_{mean} on a pixel-by-pixel basis to generate the colour maps showed in Figure 2B and S1A. Tables S1-S4 show the full reports for the fitting of each dataset.

For the time series experiments (Figure 4D,S9), the fraction of *holo*-protein from the total population of mAPXmEGFP in cells, f_{holo}, was used to indicate the change in heme bioavailability. The change in f_{holo} at each time point (t_i) relative to that observed at t_0, was converted to a percentage value, Equation S2:

$$\%\Delta f_{holo} = [f_{(holo,\, ti)} - f_{(holo,\, t0)}]*100 \qquad \text{...Eq. S2}$$

By combining the photon counting histograms measured across all the pixels in an image of HEK293 cells, the overall amplitudes α_{fast} and α_{slow} for the fluorescence decay components of mAPXmEGFP at each time point was obtained. The values of α_{fast} and α_{slow} can be used to find $f_{(holo)}$ according to Equation S3.

$$f_{(holo)} \propto \alpha_{fast}/(\alpha_{fast} + \alpha_{slow}) \qquad \text{...Eq. S3}$$

where the constant of proportionality, α, in Equation S3 for mAPXmEGFP was obtained in previously published work (*27*).

mAPXmEGFP heme sensor plasmids and sequences. For the expression of mAPXmEGFP in HEK293 cells a pLEICS-138 vector was used, Figure S2A. For the expression of mAPXmEGFP in *E. coli* a pLEICS-45 vector was used, Figure S2B. Cloning of the mAPXmEGFP sequence into the pLEICS-138 and pLEICS-45 vectors was carried out by PROTEX (University of Leicester). The sequence of the insert cloned into pLEICS-45 (completed with His-Tag for recombinant purification) is pasted below:

ATGCACCATCATCATCATCATCCCGGGATGGTGAGCAAGGGCGAGGAGCTGTTCACCGGGGTG
GTGCCCATCCTGGTCGAGCTGGACGGCGACGTAAACGGCCACAAGTTCAGCGTGTCCGGCGAG
GGCGAGGGCGATGCCACCTACGGCAAGCTGACCCTGAAGTTCATCTGCACCACCGGCAAGCTG
CCCGTGCCCTGGCCCACCCTCGTGACCACCCTGACCTACGGCGTGCAGTGCTTCAGCCGCTAC
CCCGACCACATGAAGCAGCACGACTTCTTCAAGTCCGCCATGCCCGAAGGCTACGTCCAGGAG
CGCACCATCTTCTTCAAGGACGACGGCAACTACAAGACCCGCGCCGAGGTGAAGTTCGAGGGC
GACACCCTGGTGAACCGCATCGAGCTGAAGGGCATCGACTTCAAGGAGGACGGCAACATCCTG
GGGCACAAGCTGGAGTACAACTACAACAGCCACAACGTCTATATCATGGCCGACAAGCAGAAGA

ACGGCATCAAGGTGAACTTCAAGATCCGCCACAACATCGAGGACGGCAGCGTGCAGCTCGCCG
ACCACTACCAGCAGAACACCCCCATCGGCGACGGCCCCGTGCTGCTGCCCGACAACCACTACC
TGAGCACCCAGTCCAAACTGAGCAAAGACCCCAACGAGAAGCGCGATCACATGGTCCTGCTGGA
GTTCGTGACCGCCGCCGGGATCACTCTCGGCATGGACGAGCTGTACAAGTCCGGACTCAGATC
TGGCGGCGGCGGAAAGTCTTACCCAACTGTGAGTGCTGATTACCAGGACGCCGTTGAGAAGGC
GAAGAAGAAGCTCAGAGGCTTCATCGCTGAGAAGAGATGCGCTCCTCTAATGCTCCGTTTGGCA
TGGCACTCTGCTGGAACCTTTGACAAGGGCACGAAGACCGGTGGACCCTTCGGAACCATCAAGC
ACCCTGCCGAACTGGCTCACAGCGCTAACAACGGTCTTGACATCGCTGTTAGGCTTTTGGAGCC
ACTCAAGGCGGAGTTCCCTATTTTGAGCTACGCCGATTTCTACCAGTTGGCTGGCGTTGTTGCC
GTTGAGGTCACGGGTGGACCTAAAGTTCCATTCCACCCTGGAAGAGAGGACAAGCCTGAGCCAC
CACCAGAGGGTCGCTTGCCCGATGCCACTAAGGGTTCTGACCATTTGAGAGATGTGTTTGGCAA
AGCTATGGGGCTTACTGACCAAGATATCGTTGCTCTATCTGGGGGTCACACTATTGGAGCTGCA
CACAAGGAGCGTTCTGGATTTGAGGGTCCCTGGACCTCTAATCCTCTTATTTTCGACAACTCATA
CTTCACGGAGTTGTTGAGTGGTGAGAAGGAAGGTCTCCTTCAGCTACCTTCTGACAAGGCTCTTT
TGTCTGACCCTGTATTCCGCCCTCTCGTTGATAAATATGCAGCGGACGAAGATGCCTTCTTTGCT
GATTACGCTGAGGCTCACCAAAAGCTTTCCGAGCTTGGGTTTGCTGATGCCTAA

The amino acid sequence of mAPXmEGFP is shown below (**green**: mEGFP; **red**: mAPX):
**MVSKGEELFTGVVPILVELDGDVNGHKFSVSGEGEGDATYGKLTLKFICTTGKLPVPWPTLVTTLTY
GVQCFSRYPDHMKQHDFFKSAMPEGYVQERTIFFKDDGNYKTRAEVKFEGDTLVNRIELKGIDFKE
DGNILGHKLEYNYNSHNVYIMADKQKNGIKVNFKIRHNIEDGSVQLADHYQQNTPIGDGPVLLPDNH
YLSTQSKLSKDPNEKRDHMVLLEFVTAAGITLGMDELYK**SGLRSGGGG**KSYPTVSADYQDAVEKA
KKKLRGFIAEKRCAPLMLRLAWHSAGTFDKGTKTGGPFGTIKHPAELAHSANNGLDIAVRLLEPLK
AEFPILSYADFYQLAGVVAVEVTGGPKVPFHPGREDKPEPPPEGRLPDATKGSDHLRDVFGKAMG
LTDQDIVALSGGHTIGAAHKERSGFEGPWTSNPLIFDNSYFTELLSGEKEGLLQLPSDKALLSDPVF
RPLVDKYAADEDAFFADYAEAHQKLSELGFADA**

Expression and purification of apo-mAPXmEGFP in E. coli. The expression and purification of apo-mAPXmEGFP was conducted as previously described (2). Briefly, an expression construct based on a pLEICES45 vector provided by PROTEX (University of Leicester, Figure S2B) was used to transform in *E. coli* BL21(DE3) cells. A starter culture was grown overnight in LB medium supplemented with 100 µg/mL ampicillin (Sigma Aldrich) and 1 mM SA at 37 °C in an orbital shaker (150 rpm). The starter culture was diluted 1:100 with LB medium supplemented with 100 µg/mL ampicillin and 1 mM SA in 2 L baffled Erlenmeyer flasks and re-incubated (37 °C, 150 rpm) until the optical density at 600 nm was between 0.4-0.7. Expression was induced with 0.5 mM isopropyl-D-1-thiogalactopyranoside (IPTG) and by dropping the temperature to 23 °C. Cells were harvested 20 h post-induction (10 minutes, 5000 g 4 °C) and resuspended in lysis buffer (10 mM KH_2PO_4, 150 mM KCl, pH = 7) supplemented with deoxyribonuclease I (Sigma Aldrich), EDTA-free protease inhibitor cocktail (Roche), and lysozyme (Sigma Aldrich). The suspension was sonicated on ice (4 bursts, 200 W, 30 seconds on/off cycles) and the lysate was clarified by centrifugation (150000 g, 30 minutes, 4 °C). The homogenate was filtered with a 0.2 µm pores syringe filter (Corning, cls431219), concentrated using a 30 kDa cut-off centrifugal filter (Millipore, UFC9030), and loaded onto a nickel affinity column (HisTrap excel 5 mL; washing buffer: [KH_2PO_4] = 10 mM, [KCl] = 150 mM, [Imidazole] = 100 mM, pH = 7; elution buffer: [KH_2PO_4] = 10 mM, [KCl] = 150 mM, [Imidazole] = 500 mM, pH = 7; T = 6 °C). The eluate was de-salted with a PD10-G25 column (GE Healthcare) following the manufacturer's protocol and further purified by gel filtration using a HiLoad Superdex 16/600 200 pg column (GE Healthcare) (elution buffer: [KH_2PO_4] = 10 mM, [KCl] = 150 mM, pH = 7; T = 6 °C). The UV-vis spectra of mAPXmEGFP and the gel filtration elution profile are shown in Figure S1C-D.

Lysis of HEK293 cells. Cells were cultured in a 6-well plate (Greiner Bio-one) or 10 cm dishes (Nunc). Before lysis, cells were gently washed with 1×PBS buffer (Greiner Bio-one). Ice-cold lysis buffer (1×RIPA buffer, ThermoFisher) supplemented with EDTA-free protease inhibitor tablet (SLS) was

added to the cells which were subsequently scraped, transferred onto a 15 mL tube and incubated on ice for 10 minutes. The cell suspension was sonicated (30-50% amplitude, 180 W, 1 burst of 2 seconds). Lysates were incubated again on ice for 10 minutes on a rocking shaker and then clarified by centrifugation (30 min, 17000 g, 4°C). Total protein in cell lysate was quantified by Pierce BCA protein assay in a 96-well plate (Greiner) following the manufacturer's protocol (Thermofisher) and using a CLARIOstar Plus fluorescence microplate reader. Lysates were aliquoted and stored at -80°C.

Immunoblotting. Typically, 5-25 µg of total protein isolated from cell lysate was prepared in a final volume of 10 µL of loading buffer using 4 x Laemmli buffer (250 mM Tris pH 6.8, 8% SDS, 40% glycerol, 1.4 M mercaptoethanol, 0.02% bromophenol blue). Samples were boiled at 100 °C for 5 minutes. Samples were loaded onto a 17-well NuPAGE 4-12% BisTris gel (11.0 mm x 17 well (Invitrogen)) and electrophoresed at a constant voltage of 160 V for 1 h. Gels were transferred onto a low-fluorescence PVDF membrane (pore size 0.2 uM; Thermo Scientific 22860) using the XCell II Blot module (Invitrogen) for a wet transfer at a constant voltage of 30 V for 1 h. Membranes were blocked with 5% Blotto dry milk in Tris Buffered Saline-Tween, TBST (0.1% Tween) for 1 h at room temperature. ALAS-1, HO-1, HO-2, GAPDH and mAPXmEGFP were separately probed by overnight primary antibody incubation at 4 °C using rabbit anti-ALAS-1 (1:1500 dilution, A8855/A32282, antibodies.com), rabbit anti-HO-1 (1:2500 dilution, A303-662A, Bethyl Laboratories), rabbit anti-HO-2 (1:2000 dilution, A305-354A, Bethyl Laboratories), rabbit anti-GAPDH (1:20000 dilution, A85377, antibodies.com), and rabbit anti-GFP (1:1000, A87774, antibodies.com). Each detection was accompanied with the measurement of β-actin as an internal control, which was probed with a 1:2000 dilution of mouse anti-actin (A85272, antibodies.com). Following primary antibody incubation, membranes were washed three times with TBST on a rocking shaker (5-minutes wash each). Membranes were incubated and gently rocked for 1 h at room temperature with goat anti-mouse secondary antibody (20344, Biotium) conjugated to a 680 nm fluorophore and donkey anti-rabbit secondary antibody (20065, Biotium) conjugated to a 790 nm fluorophore. Secondary antibodies were diluted 1:20000 in blocking buffer. After secondary antibody incubation membranes were washed three times with TBST on a rocker (5 min wash each). Membrane images were acquired using a Licor Odyssey Fc imager with 2 min acquisition on the 700 and 800 channels. Images were analysed using Empiria Studio® Software.

Determination of mAPXmEGFP concentration in HEK293. Using increasing amounts of purified mAPXmEGFP, a calibration curve for the estimation of the sensor concentration in whole lysates of HEK293 expressing mAPXmEGFP by immunoblotting was obtained (Figure S3). A total of 2.5 million cells were lysed as detailed above and the volume of each cell was assumed equal to 3 pL (3). The average cellular concentration of the sensor was determined by densitometry in the sub micromolar range (estimated 80 nM).

Analysis of total heme content. HEK293 cells were cultured in MEM α (Gibco) containing 10% FBS (Gibco), 1% (v/v) penicillin-streptomycin (Gibco, 15140-122), and supplemented with 1 mM SA, or 10 µM hemin, or 10 µM ZnPP at 37°C and 5% CO_2 in T75 or T25 flasks (Greiner) until 70-90% confluency was reached. Cells were washed twice with PBS, trypsinised (TrypLE; Gibco), resuspended in PBS buffer, and harvested by centrifugation. Cell pellets were resuspended in PBS and cells were counted. A minimum of 10^6 cells were harvested by centrifugation (300 g, 5 minutes) and the supernatant was carefully removed. Cell pellets were immediately processed to determine total heme content or stored at -80 °C for future use. Total heme content was measured fluorometrically by removing iron from heme with a saturated solution of oxalic acid and quantifying total protoporphyrin IX fluorescence against a calibration curve (*31*). Briefly, cell pellets were resuspended with 500 µL of 20 mM oxalic acid and incubated overnight at 4°C whilst protected from light. Then, 500 µL of warm (50-70°C) 2 M oxalic acid was added to each sample. Each sample was split in two tubes with one incubated in the dark at room temperature whilst the other was boiled for 30 minutes. All samples were centrifuged at 17000 g for 2 minutes and 200 µL of the supernatant was transferred in duplicate in a black 96 well plate (Greiner, 655900). Fluorescence ($\lambda_{exc,\ range}$ = 400 ± 15 nm; λ_{em} = 620 nm) was measured using a CLARIOstar

Plus microplate reader (BMG Labtech). For each sample, the signal obtained for the aliquot incubated at room temperature was subtracted from the boiled sample. A calibration curve was used to determine total heme concentration using 0-500 nM hemin chloride standards, constructed by preparing serial dilutions of a DMSO stock. The standards were incubated/boiled as described above. An averaged heme concentration per HEK293 cell was obtained from the known number of cells lysed and by assuming 3 pL as cellular volume (3).

Proteomic analysis - TMT Labelling and high pH reversed-phase chromatography. HEK293 cells that had been incubated 24 h with 1 mM SA, 10 µM hemin, or 10 µM ZnPP were harvested and lysed as described above. Total protein content in each lysate sample was determined by BCA assay (Thermo Fischer). Aliquots of 50 µg of total protein sample were digested with trypsin (1.25 µg trypsin; 37°C, overnight), labelled with Tandem Mass Tag (TMTpro) sixteen plex reagents according to the manufacturer's protocol (Thermo Fisher Scientific, UK) and the labelled samples pooled. An aliquot of 200 µg of the pooled sample was desalted using a SepPak cartridge according to the manufacturer's instructions (Waters, Milford, Massachusetts, USA). Eluate from the SepPak cartridge was evaporated to dryness and resuspended in buffer A (20 mM ammonium hydroxide, pH 10) prior to fractionation by high pH reversed-phase chromatography using an Ultimate 3000 liquid chromatography system (Thermo Fisher Scientific). In brief, the sample was loaded onto an XBridge BEH C18 Column (130 Å, 3.5 µm, 2.1 mm X 150 mm, Waters, UK) in buffer A and peptides eluted with an increasing gradient of buffer B (20 mM Ammonium Hydroxide in acetonitrile, pH 10) from 0-95% over 60 minutes. The resulting fractions (20 in total) were evaporated to dryness and resuspended in 1% formic acid prior to analysis by nano-LC MSMS using an Orbitrap Fusion Lumos mass spectrometer (Thermo Scientific).

Proteomic analysis - nano-LC mass spectrometry. High pH reversed-phase fractions were further fractionated using an Ultimate 3000 nano-LC system in line with an Orbitrap Fusion Lumos mass spectrometer (Thermo Scientific). In brief, peptides in 1% (vol/vol) formic acid were injected onto an Acclaim PepMap C18 nano-trap column (Thermo Scientific). After washing with 0.5% (vol/vol) acetonitrile 0.1% (vol/vol) formic acid, peptides were resolved on a 250 mm × 75 µm Acclaim PepMap C18 reverse phase analytical column (Thermo Scientific) over a 150 min organic gradient, using 7 gradient segments (1-6% solvent B over 1 min, 6-15% B over 58 min, 15-32% B over 58 min, 32-40% B over 5 min, 40-90% B over 1min, held at 90% B for 6 min and then reduced to 1% B over 1 min) with a flow rate of 300 nl min^{-1}. Solvent A was 0.1% formic acid and Solvent B was aqueous 80% acetonitrile in 0.1% formic acid. Peptides were ionized by nano-electrospray ionization at 2.0kV using a stainless-steel emitter with an internal diameter of 30 µm (Thermo Scientific) and a capillary temperature of 300 °C. All spectra were acquired using an Orbitrap Fusion Lumos mass spectrometer controlled by Xcalibur 3.0 software (Thermo Scientific) and operated in data-dependent acquisition mode using an SPS-MS3 workflow. Fourier Transform Mass Spectrometry Stage 1 (FTMS1) spectra were collected at a resolution of 120 000, with an automatic gain control (AGC) target of 200 000 and a maximum injection time of 50 ms. Precursors were filtered with an intensity threshold of 5000, according to charge state (to include charge states 2-7) and with monoisotopic peak determination set to Peptide. Previously interrogated precursors were excluded using a dynamic window (60s +/-10ppm). The MS2 precursors were isolated with a quadrupole isolation window of 0.7m/z. Second Ion Trap Mass Spectrometry Stage 2 (ITMS2) spectra were collected with an AGC target of 10 000, maximum injection time of 70ms and CID collision energy of 35%. For FTMS3 analysis, the Orbitrap was operated at 50 000 resolution with an AGC target of 50 000 and a maximum injection time of 105 ms. Precursors were fragmented by high energy collision dissociation (HCD) at a normalised collision energy of 60% to ensure maximal TMT reporter ion yield. Synchronous Precursor Selection (SPS) was enabled to include up to 10 MS2 fragment ions in the FTMS3 scan.

Proteomics - data and statistical analysis - The raw data files were processed and quantified using Proteome Discoverer software v2.4 (Thermo Scientific) and searched against the UniProt Human database (downloaded January 2024: 82415 entries) using the SEQUEST HT algorithm. Peptide

precursor mass tolerance was set at 10 ppm, and MS/MS tolerance was set at 0.6 Da. Search criteria included oxidation of methionine (+15.995 Da), acetylation of the protein N-terminus (+42.011 Da) and methionine loss plus acetylation of the protein N-terminus (-89.03 Da) as variable modifications and carbamidomethylation of cysteine (+57.0214 Da) and the addition of the TMTpro mass tag (+304.207) to peptide N-termini and lysine as fixed modifications. Searches were performed with full tryptic digestion and a maximum of 2 missed cleavages were allowed. The reverse database search option was enabled, and all data were filtered to satisfy false discovery rate (FDR) of 5%.
The MS data were searched against the human UniProt database retrieved on 2024-01-24, and updated with additional annotation information on 2024-05-08. Protein groupings were determined by PD2.4, however, the master protein selection was improved with an in-house script. The script first searches UniProt for the current status of all protein accessions and updates redirected or obsolete accessions. The script further takes the candidate master proteins for each group, and uses current UniProt review and annotation status to select the best annotated protein as master protein without loss of identification or quantification quality. The protein abundances for each sample were normalised such that all samples had an equal total protein abundance, then both raw and normalised abundances were Log2 transformed to bring them closer to a normal distribution. The data were processed and statistically analysed using normalised abundances. Univariate pairwise t-tests were performed for each comparison of interest. For all comparisons, the p-value was adjusted using the Benjamini-Hochberg FDR method. Outputs for normalised abundance were exported to Excel and formatted for ease of use. Principal Component Analyses (PCAs) were calculated using the FactoMineR package, and the plotted using the ggplot2 package. Principal Components 1 and 2 were plotted against each other to give an indication of the main sources of variance, and 3 and 4 were plotted to infer any further trends for both raw and normalised abundances. For each comparison, the $-\log_{10}$(p-value) of each protein was plotted against the $\log_2$ fold change (FC) in a volcano plot (Figures 4A, 5A, and S8A). Downstream pathway analysis was conducted using Ingenuity Pathway Analysis (Qiagen IPA) based upon differentially expressed proteins ($p<0.05$) using the full dataset as a reference proteome.

MTT assay. HEK293 cells were seeded in a clear 96 well plate (Greiner). When 80% confluency was reached, the maintenance medium was refreshed (control) or refreshed and supplemented with 1 mM SA, 10 µM ZnPP, 10 µM hemin, or 1 mM SA respectively. Cells were incubated for 24 h and the MTT (3-(4,5-Dimethylthiazol-2-yl)-2,5-Diphenyltetrazolium Bromide) assay for cell viability was performed following manufacturer's protocol (Biotium, 30006). For each condition (control, 10 µM ZnPP, 10 µM hemin, or 1 mM SA respectively) 12 independent measurements were carried out.

Flow Cytometry. Using Mito Tracker Deep Red FM – a stain for active mitochondria – the relative difference in the median fluorescence intensity (MFIs) of HEK293 cells incubated 24 h with 1 mM SA, 10 µM hemin, or 10 µM ZnPP was measured. A total 1,000,000 cells were washed once in PBS, spun at 300 g for 5 minutes, then resuspended with 500 µL of Ghost Dye™ Violet 510 viability dye (Cytek biosciences, 13-0870-T100), diluted 1:5000 in PBS and incubated at 4 ˚C for 20 minutes while protected from light. Then, to label active mitochondria cells were spun again and resuspended in 500 µL of a 50 nM solution of MitoTracker™ Deep Red FM (Invitrogen, M22426) in pre-warmed maintenance medium followed by incubation for 20 minutes at 37 ˚C. After staining, the cells were fixed with 1% (w/v) paraformaldehyde (PFA) in PBS containing 1% FBS. Samples were stored at 4 ˚C and protected from light for up to a week. At least 12 h were allowed between fixation and analysis. Flow cytometry data were collected on a NovoCyte 3000 flow cytometer and NovoExpress 1.6.2 software using the AmCyan (Violet 530/30) and APC (Red 675/30) detectors. Data analysis and visualisation carried out using FlowJo v10.10 software (BD). MFIs for each condition were calculated in FlowJo using the AutoSpill algorithm (4) to calculate the compensation matrix.

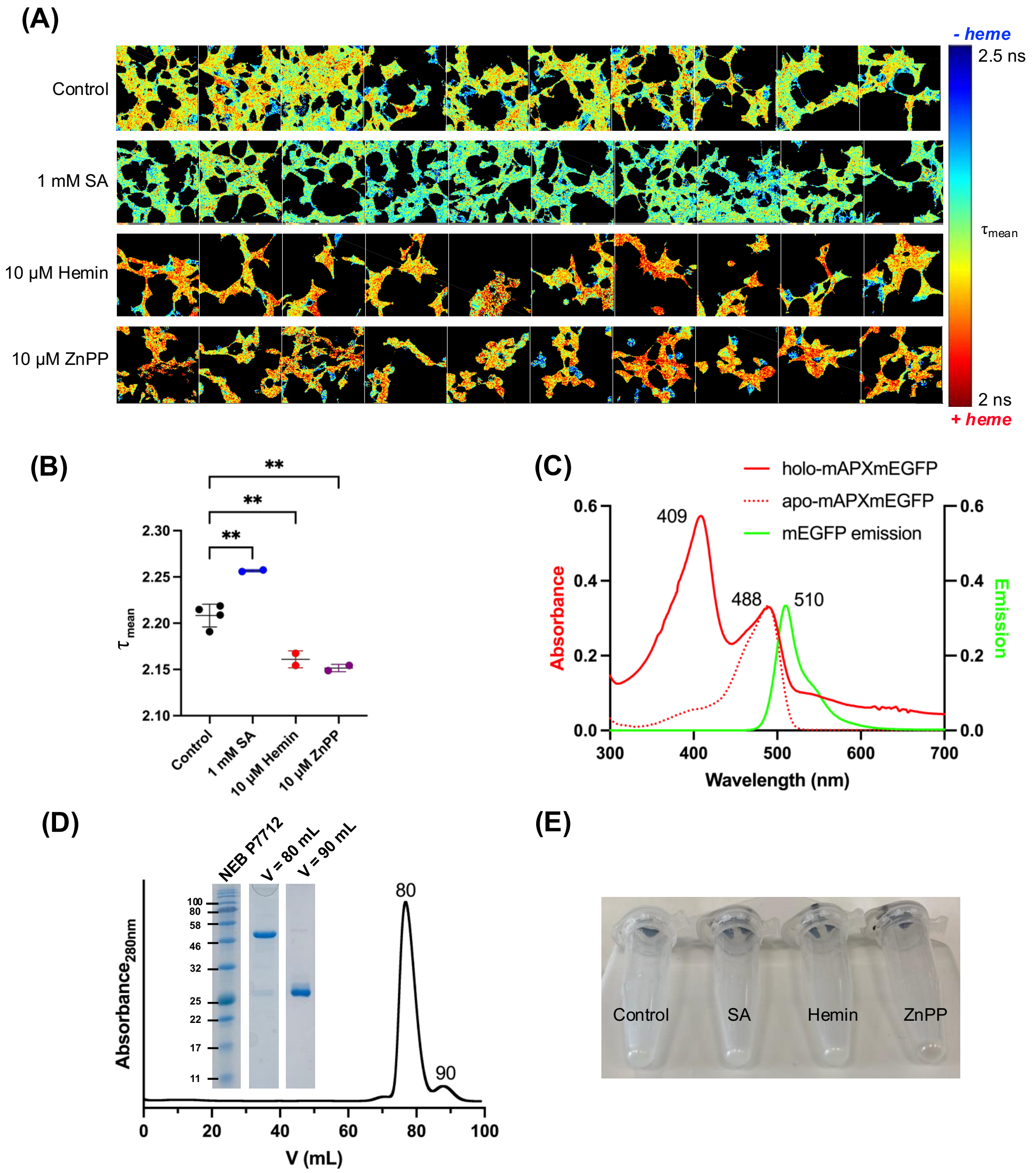

Fig. S1. Extended FLIM images of HEK293 expressing mAPXmEGFP and spectral properties of apo-mAPXmEGFP. (**A**) One set of FLIM images for HEK293 cells expressing mAPXmEGFP and incubated under normal conditions (control) and in the presence of 1 mM SA, 10 µM hemin, or 10 µM ZnPP. (**B**) Summary and comparison of the mean τ_{mean} values measured as in (A). Error bars show the standard deviation for n = 4 (Control) and n = 2 (1 mM SA, 10 µM hemin, 10 µM ZnPP). Full fitting report is available in Tables S1-4. Multiple comparisons obtained using ordinary one-way ANOVA (**: $p<0.003$). (**C**) UV-visible absorption spectra of purified apo-mAPXmEGFP (5 µM; dotted red trace), holo-mAPXmEGFP (5 µM; red trace), and emission of mEGFP (Fluorescent Protein Database) (300-700 nm). holo-mAPXmEGFP exhibits a strong heme Soret peak at 409 nm and low intensity heme Q-bands between 540 nm and 640 nm, with the mEGFP band at 489 nm. The mEGFP band is the predominant feature in the apo-mAPXmEGFP spectrum. The emission band for mEGFP overlaps with the Q-bands for holo-mAPXmEGFP. (**D**) Gel-filtration elution profile of mAPXmEGFP; HiLoad™Superdex™200 pg column (GE Healthcare). Elution buffer: [KH_2PO_4] = 10 mM, [KCl] = 150

mM, pH = 7; T = 6 °C. A first group of aggregates is followed by the peak corresponding to mAPXmEGFP (at V = c. 80 mL) and mEGFP (V = 90 mL). The SDS-PAGE gels of fractions corresponding to these latter peaks are shown in the inset. (**E**) Pellets of 1×10^6 HEK293 cells harvested after 24 h incubation in the presence of 1 mM SA, 10 μM hemin, or 10 μM ZnPP. Cells treated with ZnPP are visibly darker, consistently with tetrapyrrole accumulation as measured by total heme assay in Figure 2C.

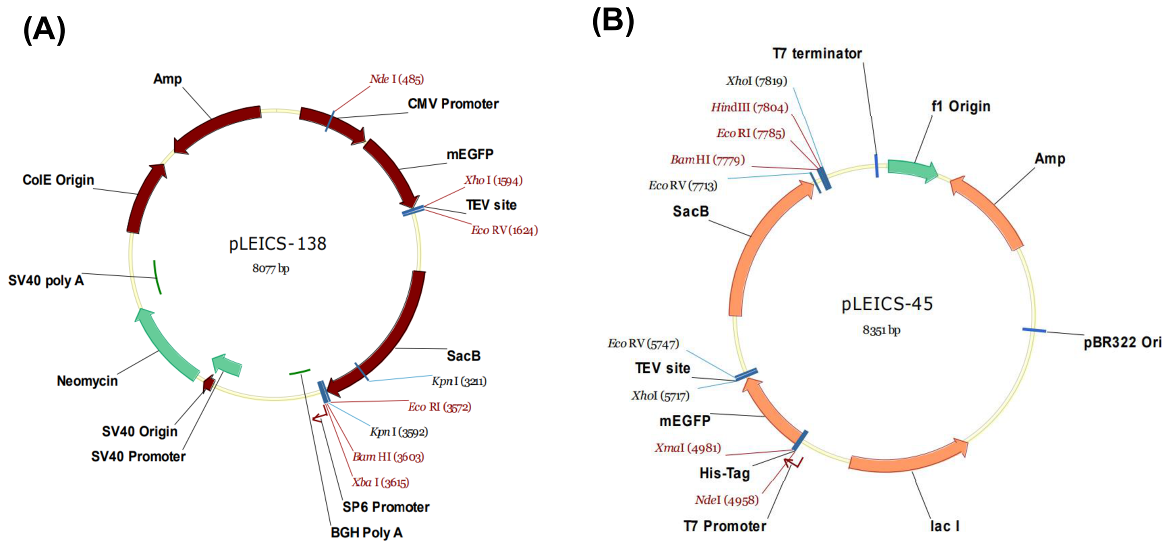

Fig. S2. Vectors used for the expression of mAPXmEGFP. (**A**) In HEK293. (**B**) In *E. coli.*

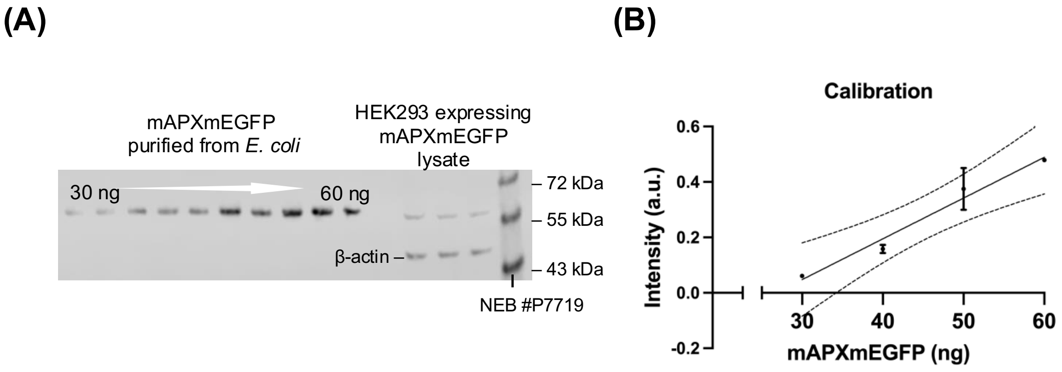

Fig. S3. Determination of [mAPXmEGFP] in a single HEK293 cell. (**A**) Increasing amounts of purified mAPXmEGFP, following overproduction in E. coli, were loaded in replicates on a SDS-PAGE gel ranging from 30 ng to 60 ng of total protein alongside 5 µg of total cell lysate obtained from 2.5 million HEK293 cells expressing mAPXmEGFP (total protein measured by BCA assay). Protein bands were transferred on a low-fuorescence PVDF membrane and band intensities detected by fluorescence immunoblotting using anti-GFP antibody (see SI text for further details). (**B**) Band intensities from the purified sensor were analysed by densitometry to build a calibration curve (y = 0.014x0.37 – 0.4; R2 = 0.98; 95% confidence interval shown) that was then used to calculate the estimated average concentration of mAPXmEGFP in HEK293 expressing mAPXmEGFP using the intensities of the bands obtained from the whole cell lysates in panel (A). The concentration of mAPXmEGFP was estimated ~ 80 nM per cell.

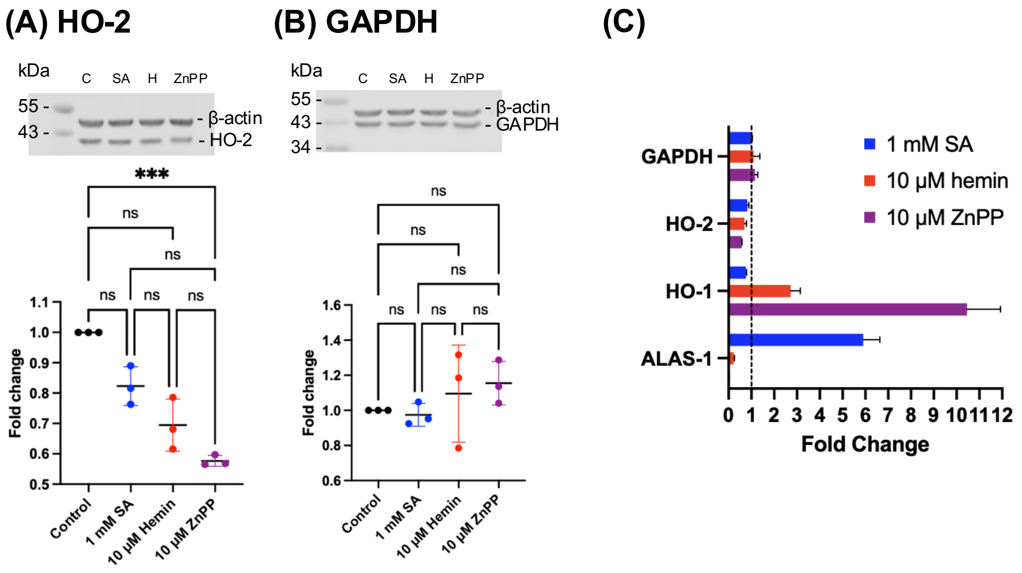

Fig. S4. Immunoblots probing the effect of 1 mM SA, 10 µM hemin, or 10 µM ZnPP on HO-2 and GAPDH levels. (**A**) The relative levels of the constitutive HO-2 protein were determined to be non-significant (ns). Multiple comparisons obtained using one-way ANOVA Tukey's test (p=***0.0004) in the presence of SA and hemin but appeared down-regulated following incubation with ZnPP. However, this observation could not be verified by quantitative proteomics. (**B**) GAPDH levels were unaffected under all conditions. (**C**) Bar chart showing the changes in the protein levels of GAPDH and HO-2; the data for HO-1 and ALAS-1 (as presented in Figure 2C) is presented alongside.

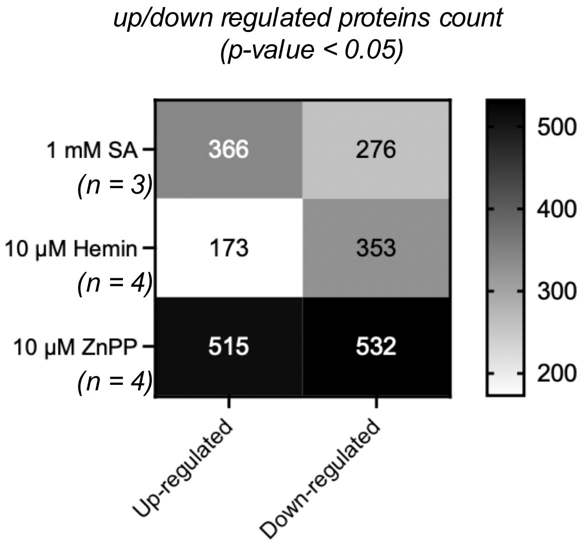

Fig. S5. Count of significantly regulated proteins measured by TMT-proteomics. Protein count of up-regulated and down-regulated proteins for each condition of 1 mM SA, 10 mM hemin and 10 mM ZnPP, with p-value < 0.05. The scale shows the relative proportions of proteins that are either up-regulated (left) or down-regulated (right) in each of the incubations.

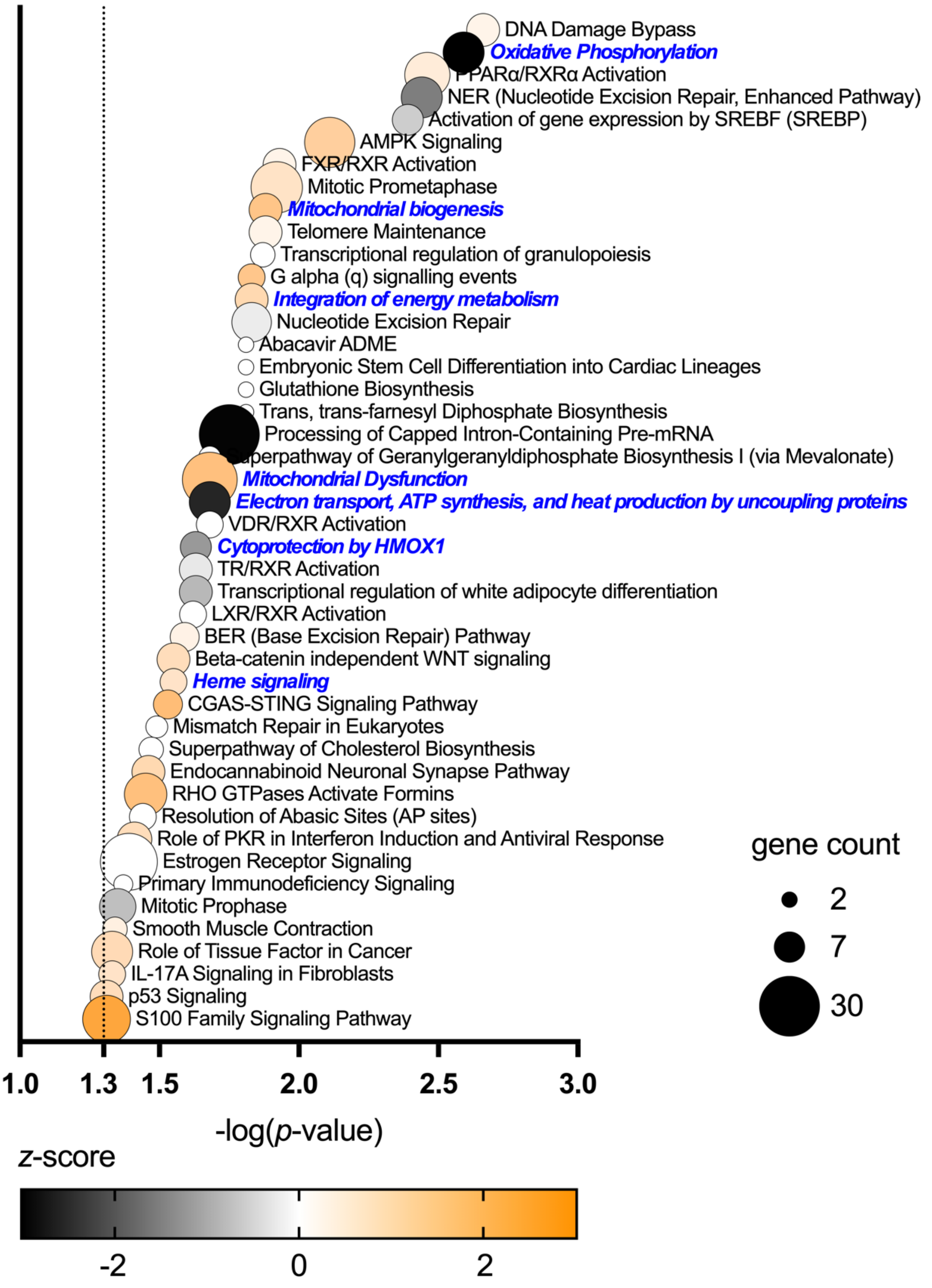

Fig. S6. Complete list of biological pathways affected by the inhibition of heme biosynthesis. Ingenuity Pathway Analysis (IPA) was applied to the TMT-proteomics data shown in Figure 4A. Pathways directly related to mitochondrial processes or heme biology are shown in blue. Pathways discussed in the manuscript that are directly related to heme biology (cytoprotection by HMOX1, heme signaling), energy metabolism (oxidative phoshoprylation, integration of energy metabolism, and electron transport, ATP synthesis, and heat production by uncoupling of proteins), or mitochondrial processes (mitochondrial biogenesis, mitochondrial dysfunction) are shown in blue.

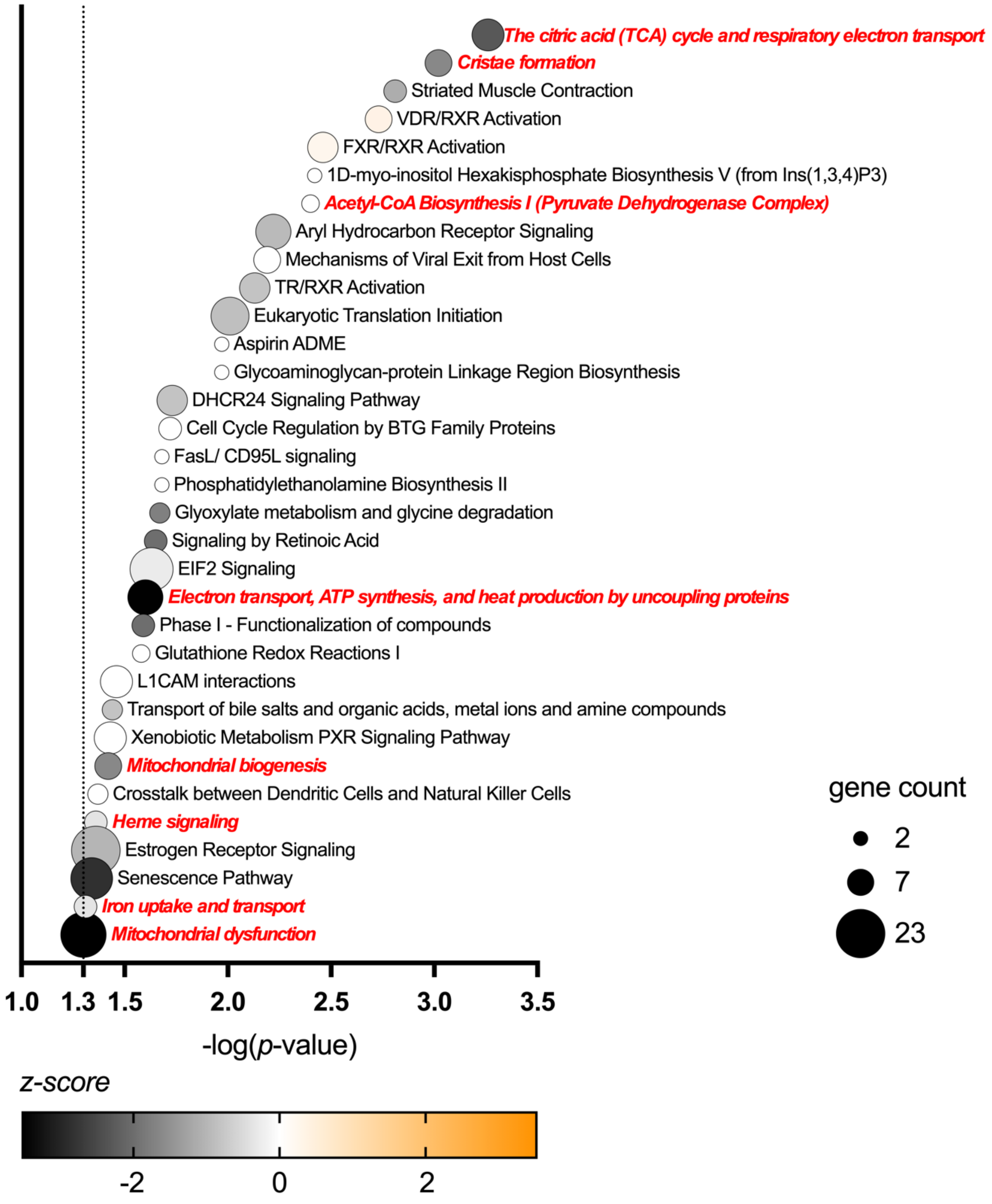

Fig. S7. Complete list of biological pathways affected by incubation with 10 μM hemin. Ingenuity Pathway Analysis (IPA) was applied to the TMT-proteomics data shown in Figure 5A. Pathways directly related to mitochondrial processes or heme biology are shown in red. Pathways discussed in the manuscript that are directly related to heme biology (heme signaling and iron uptake and transport), energy metabolism (the citric acid (TCA) cycle and respiratory electron transport, acetyl CoA biosynthesis I (pyruvate dehydrogenase complex), and electron transport, ATP synthesis, and heat production by uncoupling of proteins), or mitochondrial processes (cristae formation, mitochondrial biogenesis, and mitochondrial dysfunction) are shown in red.

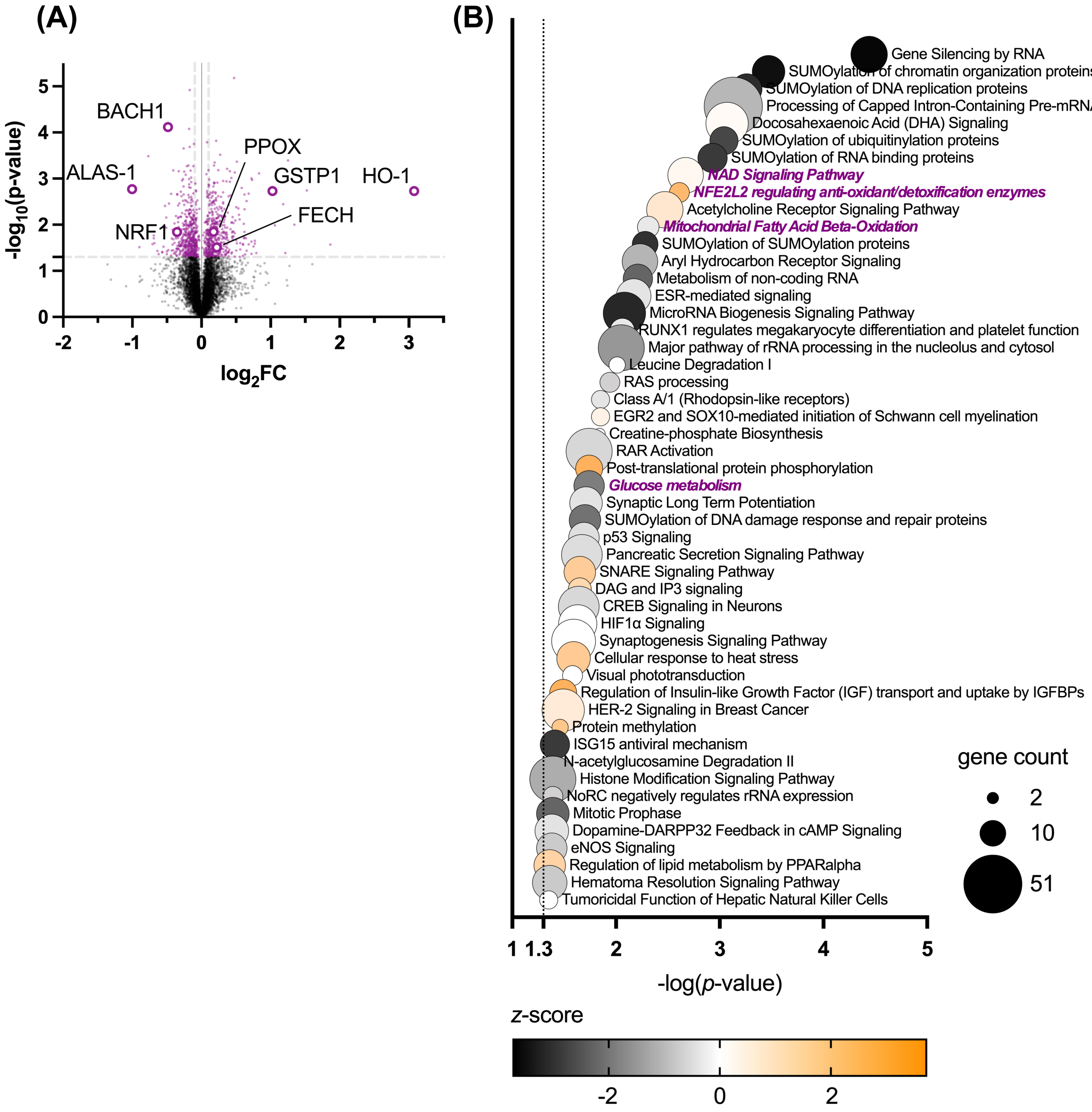

Fig. S8. The effects of incubating cells with 10 μM ZnPP on heme biosynthesis, degradation, protein expression and post-translational regulation. (**A**) Volcano plot showing the global changes in the protein levels in HEK293 cells incubated for 24 h with 10 μM ZnPP relative to whole cell lysates of control cells. The $-\log_{10}$(p-value) of each protein was plotted against the $\log_2$(FC) (FC: fold change). Differentially expressed proteins with p-value < 0.05 are shown in purple. The down-regulation of the transcriptional repressor BACH1 and the activation of NRF2-target genes (among the primary regulators of HO-1) (5, 6) determined the up-regulation of detoxifying enzymes such as HO-1 and GSTP1. However, the inhibitory effect of ZnPP on heme oxygenase activity by ZnPP opposes the detoxifying effects of HO-1 up-regulation and decreases bilirubin production (7-9) – a key antioxidant tetrapyrrole derived from heme degradation – and ultimately impairs the amelioration of oxidative stress. The regulation of the heme biosynthesis enzymes ALAS-1, protoporphyrinogen oxidase (PPOX), and ferrochelatase (FECH) is also highlighted. (**B**) Bubble plot of the biological pathways affected by the incubation with with 10 μM ZnPP. The pathway analysis was performed by Ingenuity Pathway Analysis (IPA) for the TMT-proteomics data shown in (A). Assigned *z*-scores are indicated by the colour scale underneath the bubble plot. For clarity, only pathways for which -log(*p*-value) >1.5 are shown. Pathways discussed in the manuscript are shown in purple. Major pathways involved in gene expression

regulation, chromatin and protein modifications, signalling, and responses to cellular stress were identified.

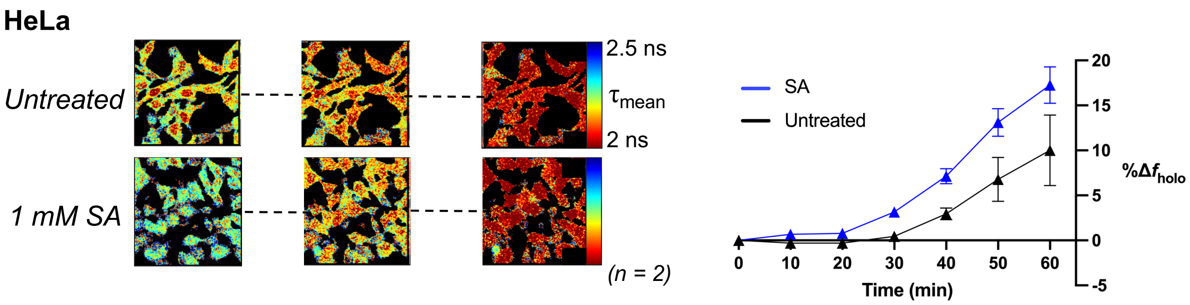

Fig. S9: Measurement of extracellular heme uptake in HeLa cells. Time-series FLIM images of clusters of HeLa expressing mAPXmEGFP acquired over 60 minutes after addition of 10 μM hemin at t_0. The decreasing τ_{mean} of mAPXmEGFP over time indicates real-time heme uptake, with warmer colours showing higher ratios of intracellular holo-mAPXmEGFP. The experiment was carried out on untreated cells (top row) and cells incubated 24h with 1 mM SA before imaging (bottom row). The plot on the right-hand side shows the percentage change of the fraction of holo-mAPXmEGFP (%Δf_{holo}). Error bars show the standard deviation for n = 2. A HeLa cell line for the stable expression of mAPXmEGFP was obtained as outlined in the Methods. The comparison of HEK293 (see 4D) and HeLa highlights different propensities for extracellular heme uptake, as this will be driven by differing demands for heme in energy production, signaling, or other cellular processes in different types of tissue. This reflects distinct metabolic profiles (e.g. compared to HEK293 cells, the higher reliance of HeLa cells on aerobic glycolysis over oxidative phosphorylation and the upregulated biosynthesis of biomolecules to support rapid cell division). The higher reliance on heme uptake measured in HeLa cells can be explained based on the involvement of dedicated heme transporters such as HRG-1. It is worth noting that differences between intracellular and extracellular heme concentrations, or rather, chemical potential, may also provide a justification for increased heme uptake when heme biosynthesis is inhibited because it would be encouraged by a greater thermodynamic gradient.

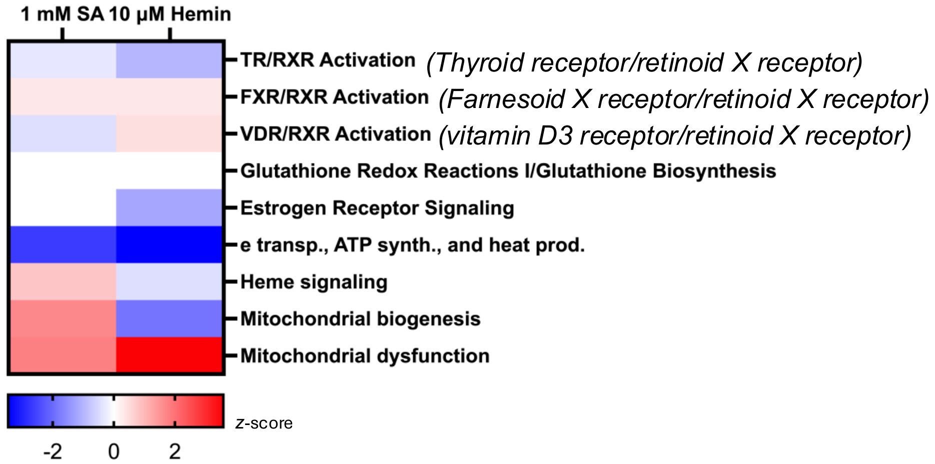

Fig. S10: Pathways significantly and independently affected by both 10 µM hemin and 1 mM SA, respectively. Heat map comparing the effects (activation/inactivation; according to z-score measured through IPA) on the biological pathways that were found significantly and independently altered by inhibition of heme biosynthesis (1 mM SA) or addition of hemin (10 µM hemin). Only pathways significantly affected by both incubations are shown.

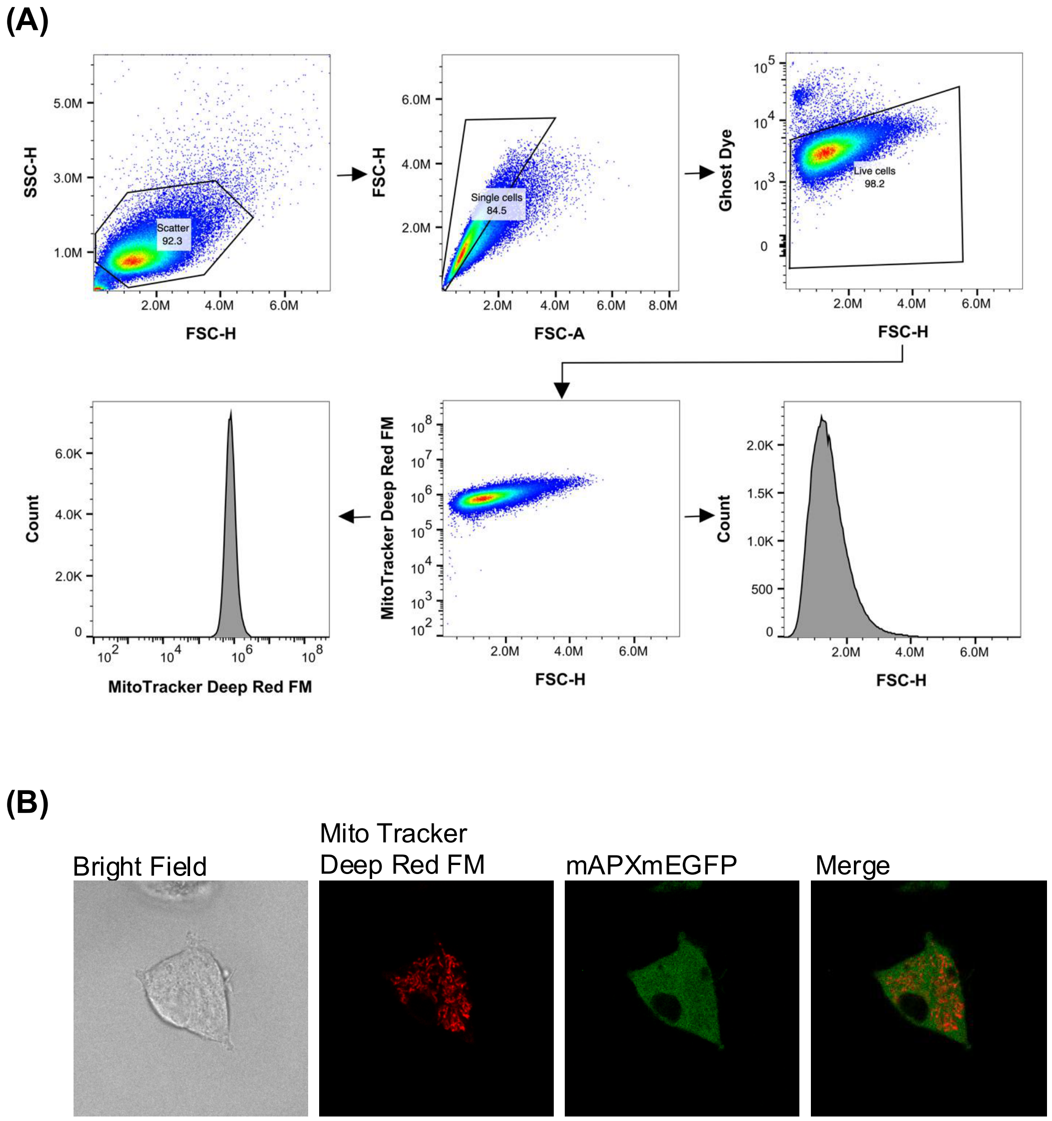

Fig. S11: Flow cytometry gating hierarchy and Mito Tracker Deep Red FM staining. (**A**) The figure shows the gating hierarchy applied to flow cytometry data. The scattering obtained for a sample of untreated cells is used as a representative example. Before fixation and analysis, cells were stained with Ghost dye to identify and exclude dead cells, and MitoTracker Deep Red FM to analyse fluorescence emission from active mitochondria. Experimental details are provided in the SI text. (**B**) Confocal images of a HEK293 cell expressing mAPXmEGFP and stained with MitoTracker Deep Red FM. Images were taken on a Leica SP5II confocal microscope using a 63×/1.20 water immersion lens and processed in FiJi. From left to right: bright-field; MitoTracker Deep Red FM (λ_{Exc} = 641 nm; λ_{Em} = 649-709 nm); mAPXmEGFP (λ_{Exc} = 488, λ_{Em} = 505-530 nm); and merge.

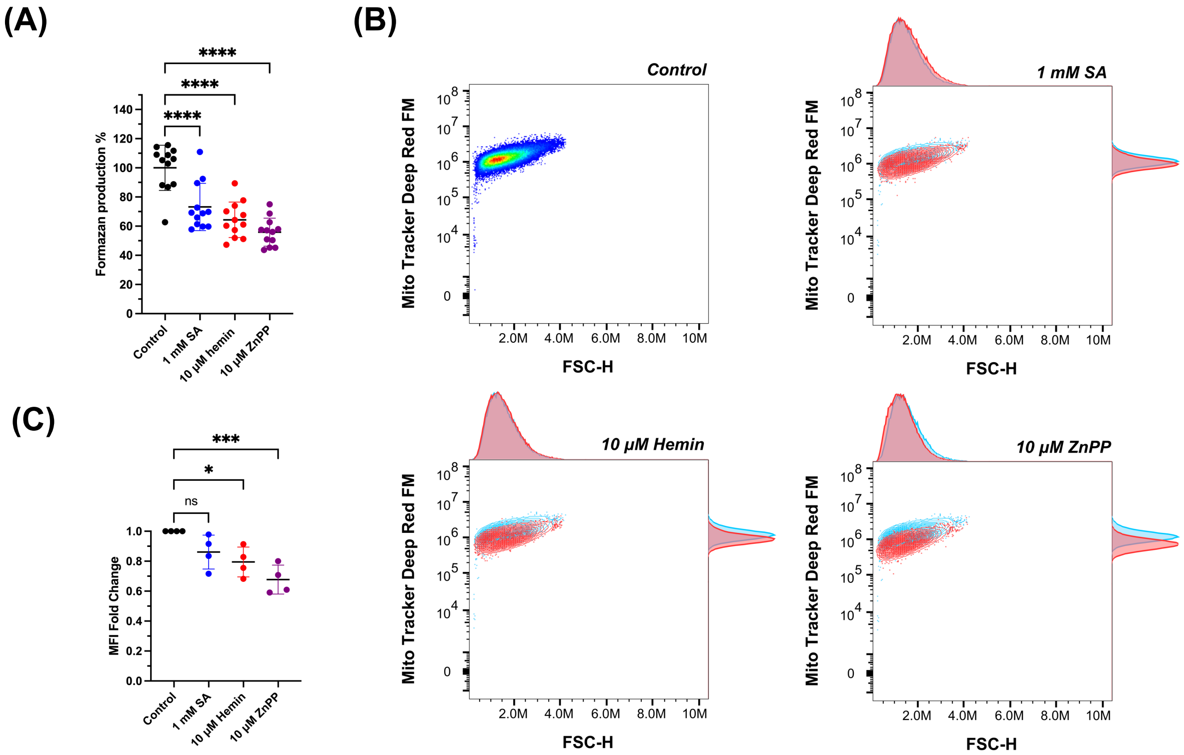

Fig. S12. Assessment of mitochondrial function and mitochondrial mass. (**A**) The chart shows the relative percentage change in metabolic activity assessed by MTT assay and reported as relative percentage change in formazan production in HEK293 cells incubated for 24 h in the presence of 1 mM SA, 10 µM hemin, or 10 µM ZnPP. Data were normalized for assays carried out on a control (no additions of SA, hemin or ZnPP to media) in each set of replicates. Error bars indicate the standard deviation over independent measurements (n = 12, ****: p<0.0001; ordinary one-way ANOVA). Assay details are provided in the SI. (**B**) Flow cytometry scatter plot representing forward scatter (FSC-H) vs fluorescence intensity of Mito Tracker Deep Red FM. Each panel shows results for single live cells isolated during data analysis by applying the gating hierarchy shown in Figure S11A. For panels showing cells incubated with 1 mM SA, 10 µM hemin, or 10 µM ZnPP the contour plots of each distribution of fluorescence intensity (red) are overlapped to data relative to the control (cyan). To highlight differences between FSC-H and Mito Tracker fluorescence due to each treatment relative to the control, adjunct histograms for each of the two parameters are shown in red and are overlapped to the control data in cyan. Outliers are shown as isolated dots. The panels show the results for a representative example obtained from a single data set. Scatter plots were created using FlowJo. (**C**) Differences in mitochondrial mass are evidenced by relative shifts in the Median Fluorescence Intensities (MFIs) of cells as measured in (B). A significant decrease in mitochondrial signal was measured due to the incubations with hemin and ZnPP, whereas the effect of 1 mM SA was deemed non-significant (n = 4, *: p<0.02, ***: p<0.0007; ordinary one-way ANOVA). A direct comparison of the mitochondrial genes affected by each incubation is shown in Figure S13.

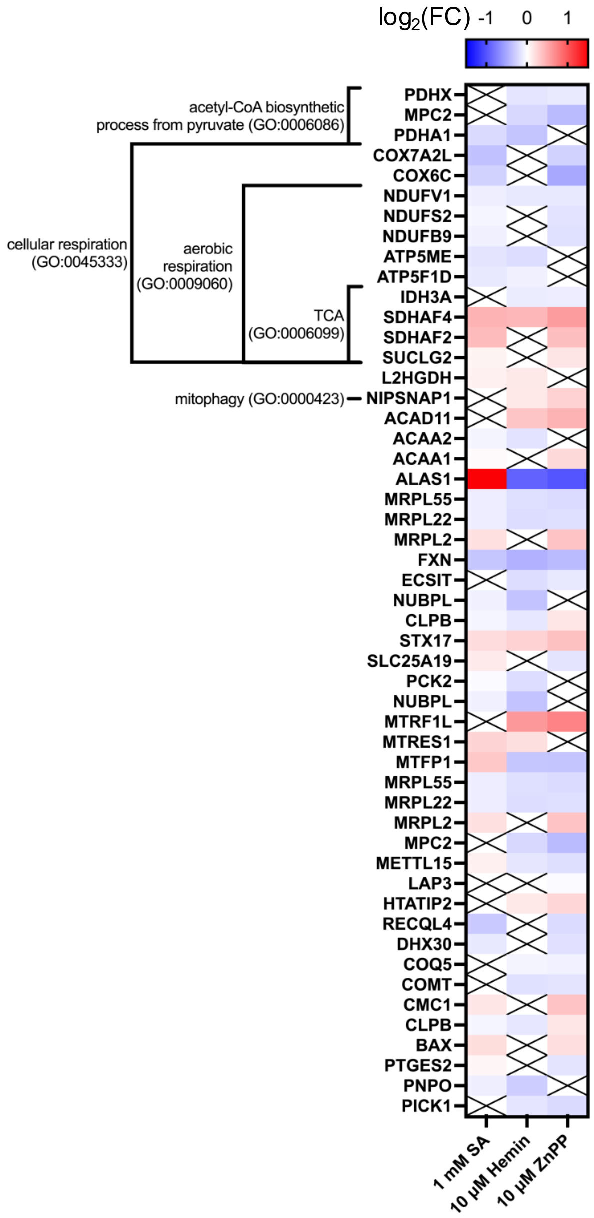

Fig. S13: Comparison of the regulation of mitochondrial genes for the conditions explored. Mitochondrial genes regulated by each of the three conditions explored were identified in the proteomics datasets through direct comparison with the Human MitoCarta 3.0. To facilitate comparison, the figure collates species that were up/down-regulated in at least 2 out of 3 datasets. Species involved in cellular respiration (GO:0045333) are indicated and relevant subclusters therein are also shown. An upregulation of protein NipSnap homolog 2 (NIPSNAP2) determined by the incubations with hemin and ZnPP - a protein that recruits the autophagy machinery required for clearance of damaged mitochondria (GO:0000423) – was identified. This is in line with flow cytometry data showing a decrease in mitochondrial mass for the incubations with hemin and ZnPP, Figure S12.

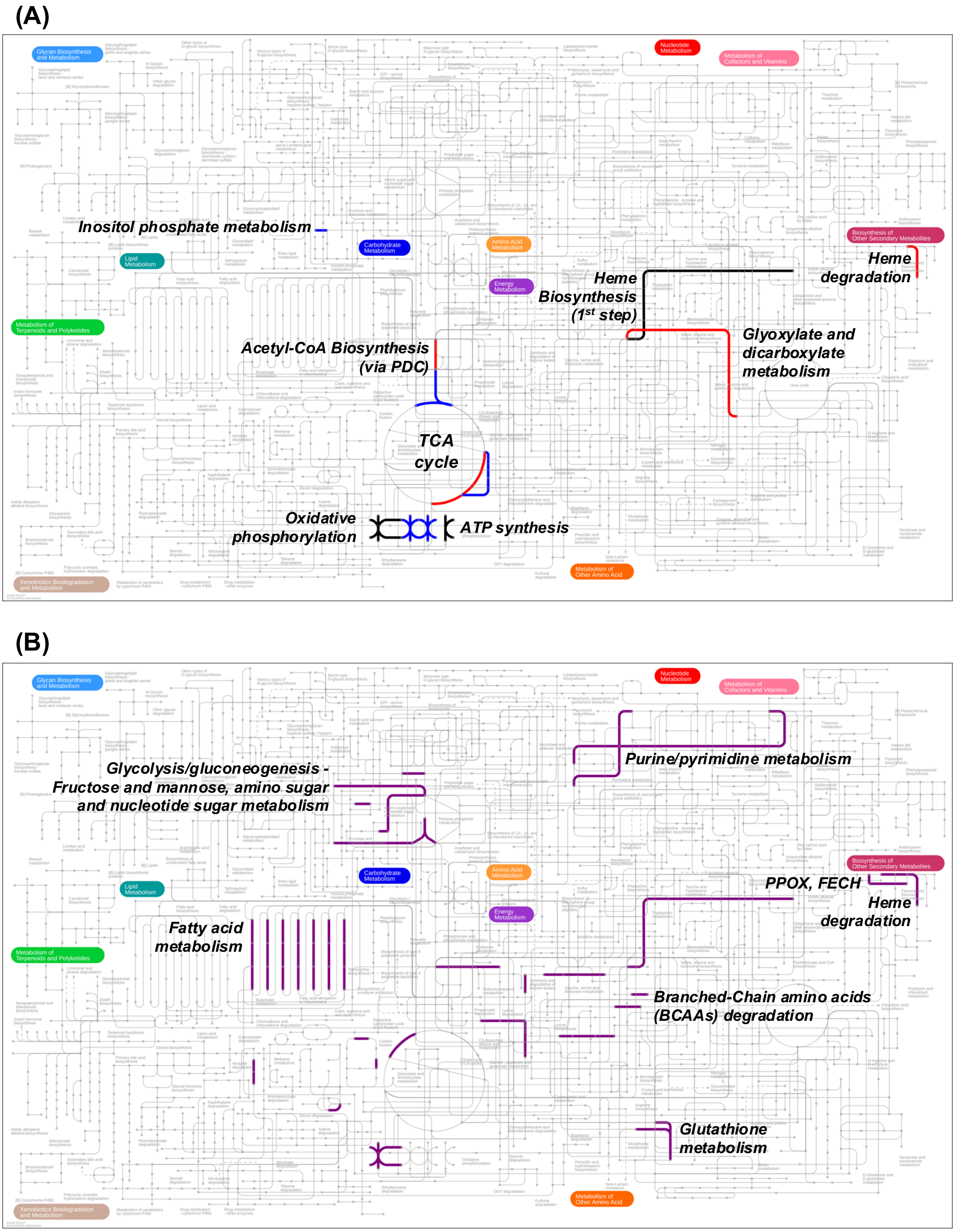

Fig. S14: Pathways identified by pathway analysis for different conditions of intracellular heme. The pathways highlighted are those belonging to the list of proteins associated with the biological pathways identified by IPA and shown in bold in Figures S6, S7 and S8B. The metabolic map was derived from the KEGG database (10) through iPath3.0 (11). Pathways are annotated for clarity. (**A**) Representation of metabolic pathways affected by low heme (incubation with SA; blue) or high heme (incubation with hemin; red). Pathways that were separately affected by both incubation with hemin and SA are shown in black. (**B**) Representation of metabolic pathways affected by incubation with ZnPP.

Table S1. Fitting parameters obtained from the FLIM images of HEK293 cells expressing mAPXmEGFP.

	Cell cluster	α_{slow}	α_{fast}	τ_{mean} (ps)	χ^2
Biological replicate #1	1	0.49095	0.50905	2244.4912	1.0071
	2	0.47631	0.52369	2222.1086	0.99197
	3	0.47625	0.52375	2220.3806	0.99721
	4	0.46162	0.53838	2199.0356	0.99392
	5	0.47611	0.52389	2225.1511	0.98966
	6	0.46436	0.53564	2206.8235	0.98682
	7	0.47516	0.52484	2218.012	0.99232
	8	0.47134	0.52866	2215.1162	1.005
	9	0.45729	0.54271	2193.0029	0.98784
	10	0.46283	0.53717	2202.6919	0.99752
Biological replicate #2	1	0.44769	0.55231	2181.3232	0.99122
	2	0.44418	0.55582	2175.114	1.0036
	3	0.46068	0.53932	2205.156	0.99847
	4	0.45598	0.54402	2194.4326	1.0004
	5	0.45477	0.54523	2192.7517	0.99248
	6	0.44999	0.55001	2185.2461	0.98668
	7	0.46181	0.53819	2201.7778	0.95872
	8	0.44968	0.55032	2185.844	0.99546
	9	0.46798	0.53202	2217.0012	0.98984
	10	0.46469	0.53531	2212.75	0.99693
	11	0.42518	0.57482	2147.7061	0.99535

Table S1: Continued from previous page.

Biological replicate #3	1	0.46362	0.53638	2203.2336	0.9945
	2	0.466	0.534	2206.8496	0.99555
	3	0.46701	0.53299	2214.7637	0.99657
	4	0.48156	0.51844	2231.9382	0.97588
	5	0.45395	0.54605	2192.4199	0.98738
	6	0.46702	0.53298	2213.771	0.99504
	7	0.45358	0.54642	2190.4011	0.97672
	8	0.46726	0.53274	2213.8372	0.99312
	9	0.46821	0.53179	2213.4089	0.98961
	10	0.46426	0.53574	2207.9524	1.0006
Biological replicate #4	1	0.46093	0.53907	2197.7898	0.98075
	2	0.47942	0.52058	2225.2678	0.98384
	3	0.47399	0.52601	2224.7344	0.97832
	4	0.47436	0.52564	2221.9263	0.98583
	5	0.47513	0.52487	2222.3469	0.98965
	6	0.46634	0.53366	2209.801	0.99434
	7	0.47836	0.52164	2228.7146	0.98199

Table S2. Fitting parameters obtained from the FLIM images of HEK293 cells expressing mAPXmEGFP and incubated for 24 h with 1mM SA.

	Cell cluster	α_{slow}	α_{fast}	τ_{mean} (ps)	χ^2
Biological replicate #1	1	0.49079	0.50921	2251.9871	0.96819
	2	0.47735	0.52265	2229.9907	0.98235
	3	0.49113	0.50887	2252.9407	0.98824
	4	0.50394	0.49606	2274.093	0.98471
	5	0.49462	0.50538	2259.5139	0.98437
	6	0.49787	0.50213	2262.7505	0.97532
	7	0.49112	0.50888	2256.0806	0.98472
	8	0.50108	0.49892	2268.3501	0.98328
	9	0.48704	0.51296	2244.8086	0.98511
	10	0.49376	0.50624	2257.0361	0.98029
Biological replicate #2	1	0.50599	0.49401	2284.9207	0.98034
	2	0.49621	0.50379	2261.7305	0.97698
	3	0.49655	0.50345	2263.2463	0.97877
	4	0.49217	0.50783	2258.8228	0.98777
	5	0.49221	0.50779	2257.2854	0.98087
	6	0.50276	0.49724	2270.2761	0.97898
	7	0.45204	0.54796	2187.9756	0.97597
	8	0.50005	0.49995	2267.0513	0.97878
	9	0.50009	0.49991	2269.4199	0.98
	10	0.49173	0.50827	2253.4985	0.97018

Table S3. Fitting parameters obtained from the FLIM images of HEK293 cells expressing mAPXmEGFP and incubated for 24 h with 10 µM Hemin.

	Cell cluster	α_{slow}	α_{fast}	τ_{mean} (ps)	χ^2
Biological replicate #1	1	0.43154	0.56846	2153.2371	1.0108
	2	0.43521	0.56479	2162.0325	0.99426
	3	0.43473	0.56527	2163.3303	0.99787
	4	0.43693	0.56307	2165.7837	0.99734
	5	0.41986	0.58014	2138.8811	0.9431
	6	0.4351	0.5649	2164.5562	0.95403
	7	0.41995	0.58005	2135.6631	1.0027
	8	0.41642	0.58358	2128.4333	0.99872
	9	0.43455	0.56545	2161.8862	1.0083
	10	0.43375	0.56625	2167.2759	0.99088
	11	0.43697	0.56303	2169.5454	1.0017
	12	0.42279	0.57721	2142.6277	0.99742
Biological replicate #2	1	0.43483	0.56517	2159.5942	1.0071
	2	0.43356	0.56644	2162.8215	0.98686
	3	0.42971	0.57029	2153.0137	0.99335
	4	0.44613	0.55387	2181.9941	1.0037
	5	0.44312	0.55688	2178.0073	0.99431
	6	0.43588	0.56412	2163.8916	0.98862
	7	0.44224	0.55776	2175.2505	0.98742
	8	0.43815	0.56185	2168.4402	0.99975
	9	0.43431	0.56569	2163.4524	0.99742

Table S4. Fitting parameters obtained from the FLIM images of HEK293 cells expressing mAPXmEGFP and incubated for 24 h with 10 µM ZnPP.

	Cell cluster	α_{slow}	α_{fast}	τ_{mean} (ps)	χ^2
Biological replicate #1	1	0.42186	0.57814	2142.2368	0.97842
	2	0.44471	0.55529	2172.1035	0.98862
	3	0.42819	0.57181	2150.7915	0.96352
	4	0.44273	0.55727	2168.9211	0.99769
	5	0.44943	0.55057	2169.9937	0.98423
	6	0.41019	0.58981	2127.1377	1.0149
	7	0.40469	0.59531	2121.3105	0.99908
	8	0.42116	0.57884	2146.7754	1.0032
	9	0.42594	0.57406	2153.1416	0.99522
	10	0.41481	0.58519	2134.5447	1.0109
Biological replicate #2	1	0.43154	0.56846	2153.2371	1.0108
	2	0.43521	0.56479	2162.0325	0.99426
	3	0.43473	0.56527	2163.3303	0.99787
	4	0.43693	0.56307	2165.7837	0.99734
	5	0.41986	0.58014	2138.8811	0.9431
	6	0.4351	0.5649	2164.5562	0.95403
	7	0.41995	0.58005	2135.6631	1.0027
	8	0.41642	0.58358	2128.4333	0.99872
	9	0.43455	0.56545	2161.8862	1.0083
	10	0.43375	0.56625	2167.2759	0.99088
	11	0.43697	0.56303	2169.5454	1.0017
	12	0.42279	0.57721	2142.6277	0.99742

Table S5. Summary table for the main intracellular effects observed and discussed in this work for each explored condition.

	1 mM SA	***10 μM Hemin***	***10 μM ZnPP***
Heme biosynthesis (ALAS-1)	up	down	down
Heme degradation (HO-1)	-	up	up
Mitochondrial processes affected	Oxidative phosphorylation, Mitochondrial biogenesis, Integration of energy metabolism, Mitochondrial dysfunction, e transport, ATP synth., and heat prod.	TCA cycle, Cristae formation, Acetyl-CoA biosynthesis, Glyoxylate mechanisms and Gly degradation, e transport, ATP synth., and heat prod.	NAD signaling pathway, NRF2 regulating anti-oxidant/detox. enzymes, Mitochondrial fatty acids β-oxidation, Glucose metabolism
Mitochondrial mass	-	down	down
Oxidative stress	-	up	up

Data S1 (Separate file). TMT-proteomics data report.

Data S2 (Separate file). Ingenuity Pathway Analysis (IPA) output for the 1 mM SA incubation.

Data S3 (Separate file). Ingenuity Pathway Analysis (IPA) output for the 10 µM hemin incubation.

Data S4 (Separate file). Ingenuity Pathway Analysis (IPA) output for the 10 µM ZnPP incubation.

References

1. S. C. Warren *et al.*, Rapid global fitting of large fluorescence lifetime imaging microscopy datasets. *PLoS One* **8**, e70687 (2013).
2. G. C. Leung *et al.*, Unravelling the mechanisms controlling heme supply and demand. *Proc Natl Acad Sci U S A* **118** (2021).
3. L. Liu, A. B. Dumbrepatil, A. S. Fleischhacker, E. N. G. Marsh, S. W. Ragsdale, Heme oxygenase-2 is post-translationally regulated by heme occupancy in the catalytic site. *J Biol Chem* **295**, 17227-17240 (2020).
4. C. P. Roca *et al.*, AutoSpill is a principled framework that simplifies the analysis of multichromatic flow cytometry data. *Nat Commun* **12**, 2890 (2021).
5. S. K. Niture, R. Khatri, A. K. Jaiswal, Regulation of Nrf2-an update. *Free radical biology & medicine* **66**, 36-44 (2014).
6. N. K. Campbell, H. K. Fitzgerald, A. Dunne, Regulation of inflammation by the antioxidant haem oxygenase 1. *Nat Rev Immunol* **21**, 411-425 (2021).
7. V. Turcanu, M. Dhouib, P. Poindron, Determination of heme oxygenase activity in murine macrophages for studying oxidative stress inhibitors. *Anal Biochem* **263**, 251-253 (1998).
8. J. E. Clark, R. Foresti, C. J. Green, R. Motterlini, Dynamics of haem oxygenase-1 expression and bilirubin production in cellular protection against oxidative stress. *Biochem J* **348 Pt 3**, 615-619 (2000).
9. T. A. Takeda, A. Mu, T. T. Tai, S. Kitajima, S. Taketani, Continuous de novo biosynthesis of haem and its rapid turnover to bilirubin are necessary for cytoprotection against cell damage. *Sci Rep* **5**, 10488 (2015).
10. M. Kanehisa, S. Goto, KEGG: kyoto encyclopedia of genes and genomes. *Nucleic Acids Res* **28**, 27-30 (2000).
11. Y. Darzi, I. Letunic, P. Bork, T. Yamada, iPath3.0: interactive pathways explorer v3. *Nucleic Acids Res* **46**, W510-W513 (2018).
